# Patterns of phosphorylated tau accumulation in a spectrum of acquired and developmental brain lesions associated with refractory epilepsy

**DOI:** 10.1111/epi.18418

**Published:** 2025-04-29

**Authors:** Alicja Mrzyglod, Anya Mebrouk, Joanna Bartkiewicz, Hanaa El Hachami, Maritchka Ryniejska, Jane de Tisi, Roland Coras, Ingmar Blumcke, Fenglai Xiao, Anna Miserocchi, Andrew McEvoy, Matthias Koepp, Joan Liu, Maria Thom

**Affiliations:** ^1^ Department of Clinical and Experimental Epilepsy UCL Queen Square Institute of Neurology London UK; ^2^ Institute of Neuropathology University Hospitals Erlangen Erlangen Germany; ^3^ School of Life Sciences University of Westminster London UK; ^4^ Division of Neuropathology University college London Queen Square Institute of Neurology London UK

**Keywords:** acquired scar, developmental, focal epilepsy, tau

## Abstract

**Objective:**

Phosphorylated tau (pTau) has been reported in surgical resections in refractory epilepsy. It is unclear whether this is activity‐driven physiological pTau or signifies the advent of neurodegenerative cascades, relevant to memory decline. To date, primarily hippocampal sclerosis and focal cortical dysplasia (FCD) type II have been studied. We aimed to explore pTau in a range of acquired and developmental epileptogenic pathologies to assess its prevalence and identify potential drivers.

**Method:**

A total of 104 cases were studied representing FCD IA (*n* = 11), FCD IIIA (*n* = 5), FCD IIIB (*n* = 6), cavernoma (*n* = 11), Sturge–Weber leptomeningeal angiomatosis (*n* = 10), meningioangiomatosis (*n* = 4), perinatal infarcts (*n* = 9), Rasmussen encephalitis (RE; *n* = 6), gray matter heterotopia (*n* = 6), old scars (*n* = 10), and temporal lobe encephaloceles (*n* = 7); we also included focal microinjuries following prior stereoelectroencephalography at different ages (*n* = 19; four in lesion‐negative cases). pTau was evaluated with AT8 immunohistochemistry, with further multiplex panels of AT8 with other established pTau markers (AT100, AT180, PHF1, CP13), pS6, glial fibrillary acidic protein, reelin, calbindin, and Tbr1 in selected cases. Labeling in the lesion was compared with adjacent cortex and clinical factors such as epilepsy duration.

**Results:**

pTau was identified in low to moderate levels in 60% overall, mainly localized to the epileptogenic lesion and more frequent in vascular malformations (74%–100%). pTau was noted in the superficial cortex across pathologies including encephaloceles, associated with superficial gliosis. In perinatal infarcts, distinct pTau patterns were noted in the superficial ulegyric cortex and heterotopic neuronal islands. Glial pTau was rare, and FCD IA, FCD IIIA/B, and microinjuries were negative. Variable regional expression of AT8 and mTOR activation markers (pS6) was noted, including in one RE case. Higher pTau expression was associated with older age at surgery and at onset of epilepsy, suggesting additional age‐related vulnerability.

**Significance:**

Our findings highlight localized and distinct patterns of pTau in some epilepsy pathologies. Plausible pathomechanisms include local vascular insufficiency, neuronal dysmaturation, and aging as well as seizure activity and provide direction for future exploration.

**Key points**

- There is interest in the role of pTau in epileptogenesis and memory impairment in patients with epilepsy.
- In varied developmental and acquired epilepsy surgical pathologies, we noted low to moderate pTau in 60%, confirming variable phosphorylation sites.
- Vascular lesions showed more frequent localized pTau, which may implicate vascular deficiency or impaired clearance mechanisms.
- Superficial cortical pTau, including in some encephaloceles, could implicate injury and gliosis.
- There was minimal cell colocalization of pTau with pS6 for mTOR pathway activation, and patterns noted in some perinatal infarcts may reflect dysmaturation.

## 1 INTRODUCTION

Phosphorylated tau (pTau) accumulation has been reported in the context of focal epilepsy, including low‐grade epilepsy‐associated tumors, focal cortical dysplasia (FCD) type II, and hippocampal sclerosis.^1^, ^2^ There is current interest surrounding the clinical significance of tau accumulation in epilepsy, in particular, whether nonaggregated tau forms enable epileptogenesis^3^, ^4^ or represent transient neuroprotective cellular responses to seizures.^5^ It is also unclear whether long‐term epilepsy represents a risk for abnormal tau aggregation and cognitive impairment as a manifestation of more common age‐related tauopathies, such as Alzheimer disease (AD), primary age‐related tauopathy, or chronic traumatic encephalopathy (CTE), or as a distinct process, akin to the idiosyncratic pTau accumulation in varied chronic inflammatory brain conditions.^6^

There are multiple potential causes of pTau accumulation in epilepsy. mTOR pathway activation is a potential candidate driver, supported by evidence of pTau in dysmorphic neurons of FCD type II and tuberous sclerosis (TS),^7^ pathologies with mTOR pathway gene activating mutations,^8^ and the ascribed role of mTOR in tau phosphorylation and neurofibrillary tangle formation in AD.^9^ As mTOR pathway activation is also a universal finding in a broad spectrum of developmental and acquired pathologies in focal epilepsy,^10^ this may represent a critical and common mechanism. Neuronal activity also stimulates tau release and its phosphorylation,^11^, ^12^ pTau is increased in experimental epilepsy models,^13^ and local seizure activity may represent a further driver.^14^ Tau is also critical to normal neurodevelopment, with fetal‐specific phosphorylation sites reported.^15^, ^16^, ^17^ As many epilepsy pathologies have neurodevelopmental origins^18^ and/or associate with early onset of seizures, observed pTau may also represent a "dysmaturational" phenomena.

A wide range of acquired as well as developmental pathologies are commonly encountered in adult epilepsy surgical practice, including vascular malformations, inflammatory pathologies, old injuries, and other FCD types.^19^ We hypothesized that exploration of the prevalence and distribution of pTau may shed light on any relationship to disease activity, abnormal neurodevelopment, regional mTOR activation, and vascular dysfunction.

## 2 MATERIALS AND METHODS

### 2.1 Case selection

A total of 104 cases were included, 83 cases from the Epilepsy Society Brain and Tissue Bank at University College London (UCL) with an additional 21 cases provided by the European Neuropathology Reference Center for Epilepsy Surgery in Erlangen, representing the range of pathologies summarized in Table 1 (case detail in Table S1).

**TABLE 1:** Clinical details and epilepsy in the pathology groups.

Characteristic	Group												Total/significance between groups
	FCD I A	FCD III A	FCD III B	Cavernoma	SWS	Meningioangiomatosis	Perinatal infarct	Rasmussen encephalitis	WM heterotopia	Cortical/WM scars	Scars from ICE	Encephalocele	
Cases, *n*	11	5	6	11	10	4	9	6	6	10	19	7	104
Age at surgery, years, mean (range) Number with data	12.7 (2–38) *n* = 11	44.1 (23–57) *n* = 5	34.6 (23–42) *n* = 6	48.3 (27–68) *n* = 11	22.78 (1–59) *n* = 10	53.9 (35–67) *n* = 4	28.2 (2–52) *n* = 8	28.12 (15–57) *n* = 6	55.67 (25–83) *n* = 6	35.7 (17–54) *n* = 10	33.97 (19–60) *n* = 19	40.82 (28–50) *n* = 7	*p* < .001
Age at onset of epilepsy, years, mean (range) Number with data	7.2 (0–34) *n* = 10	10.8 (9–13) *n* = 5	10.8 (1–32) *n* = 6	36.4 (6–61) *n* = 11	11.93 (1–40) *n* = 7	29.5 (12–67) *n* = 4	11.6 (2–18) *n* = 4	14.1 (2–21) *n* = 5	11.2 (2–22) *n* = 5	16.9 (3–35) *n* = 10	13.2 (4–30) *n* = 18	18 .00 (5–25) *n* = 7	*p* < .001
Duration, years, mean (range) Number with data	6.5 (2–16) *n* = 10	33.2 (14–45) *n* = 5	23.79 (9–37) *n* = 6	11.72 (0–34) *n* = 11	13.9 (1–51) *n* = 7	24.57 (1–40) *n* = 4	15.35 (8–35) *n* = 5	16.64 (5–36) *n* = 5	41.8 (23–80) *n* = 5	18.8 (7–45) *n* = 10	20.5 (7–44) *n* = 19	22.11 (6–29) *n* = 7	*p* < .001
Surgical/PM	11/0	5/0	6/0	11/0	10/0	4/0	6/3	4/2	0/6	10/0	19/0	7/0	Not tested
Male/female	5/5	2/3	3/3	8/3	7/3	2/2	5/4	1/5	2/4	5/5	9/10	2/5	n/s
Left/right	6/4	3/2	3/3	7/4	4/5	1/3	4/2 (2 Bilat)	2/4	Bilat	1/9	8/11	4/3	n/s
Lobar resection	O 100%	T 100%	T 100%	T 82% F 9% P 9%	T 20% F 30% P 10% O 20%	T 75% F 25%	T 22% F 11% O 56% H 11%	T 33% O 16.7% H 50%	T 33.3% F 16.7 P 33.3% H 16.7%	T 60% F 30% H 10%	T 42% F 48% P 10%	T 100%	*p* < .001
FAS	No data	60%	66.7%	55%	100%	33%	100%	75%	No data	52%	55%	71.4%	
FS	No data	100%	100%	100%	100%	100%	80%	80%	100%	100%	94%	100%	
GS^a^	No data	80%	50%	66.7%	50%	100%	80%	100%	100%	88%	94%	71%	
Episodes of SE^a^	No data	40%	16.7%	11%	0%	0%	0%	100%	100%	25%	15.8%	0%	*p* < .005
History of HI^a^	No data	40%	50%	11%	0%	66%	0%	0%	75%	22%	16%	50%	n/s
IPI^a^	No data	0%	0%	11%	0%	0%	0%	0%	0%	22%	10%	0%	n/s
Seizure‐free outcome (ILAE class 1) at 2 years	No data	60%	67%	36%	–	75%	–	33%	–	28%	32%	50%	Not tested

*Note*: Statistical differences between groups is shown with Kruskall–Wallis test and chi‐squared test for categorical variables. Duration of epilepsy for PMs is date of death rather than surgery. Brain regions resected or where lesions were located in PMs include cases where site was not specified. In some cases, the resections included multiple lobes (e.g., parieto‐occipital); for these, the major lobe resected was used as the primary location.

Abbreviations: Bilat, bilateral; F, frontal; FAS, focal aware seizures, formerly recorded as simple partial seizures; FCD, focal cortical dysplasia; FS, focal impaired awareness seizures (formerly termed complex partial seizures); GS, generalized (tonic–clonic) seizures; H, hemispherectomy; HI, history of traumatic brain or head injury; ICE, intracranial recording electrode or other injury; ILAE, International League Against Epilepsy; IPI, initiating precipitating injury of a complex febrile seizure; n/s, not significant; O, occipital; P, parietal; PM, postmortem tissue sample; SE, prior episode of status epilepticus; SWS, Sturge–Weber syndrome; T, temporal; WM, white matter.

^a^ Data are presented as percentage of all cases with data; for some variables, there was no clinical data available, so this may be an underestimate. ILAE seizure‐free outcome (class 1) is shown only shown for groups where data were available for more than half of the cases (follow‐up data were not available in recently operated cases).

Cases were initially evaluated with a broad diagnostic immunohistochemistry panel as recommended in International League Against Epilepsy guidelines.^20^ AT8 immunohistochemistry carried out on all cases was evaluated independently by three observers and a semiquantitative score was devised for the lesional area, based on region, with maximal staining as follows: score = 0, negative; 1, rare (<5); 2, few (5–50); and 3, abundant AT8^+^ neurites and/or neurons. In 74 cases, adjacent normal‐appearing nonlesional cortex was available (on the same or a further section) and AT8 similarly quantified. In view of the recognizable pathology in these cases, it was not possible to be blinded as to the underlying lesion. Further immunostaining panels were conducted on selected cases from pathology groups as detailed in Table 2 and Data S1.

Clinical data of any prior head injuries, age at onset, duration, and seizure types in addition to perinatal/birth injury were obtained. Kruskal–Wallis and chi‐squared tests were used to compare differences in clinical parameters between the pathology group and pTau semiquantitative scores, and linear regression analysis was used to compare differences in age at onset, duration, and age at surgery with pTau scores as well as postsurgical outcomes for cases with follow‐up data (see Table 1).

**TABLE 2:** Immunohistochemistry panel.

Marker	Protocol	Antibody	Source, clone	Dilution
Tau PTM (phosphorylation)	IHC (A)	AT8 (Ser202/Thr205) IHC	Thermo Fisher Scientific, MN1020	1:1200
Beta‐amyloid	IHC (S)	Amyloid‐beta	Dako, M0872	1:50
mTOR pathway activation	IHC/IF (A)	Phospho‐S6 ser235‐236	Cell Signaling Technology, 4857	1:200
mTOR pathway activation	IHC (S)	Phospho‐S6 ser240‐244	Cell Signaling Technology, 5364	1:1000
Astroglia	IHC/IF (A)	GFAP	Dako, GFAP	1:2500
Interneuronal marker	IF (S)	Calretinin	Merck, C7479	1:100
Developmental regulatory protein	IF (S)	Reelin	Merck, MAB5366	1:500
Radial migratory neuronal marker	IF (S)	Tbr1	Abcam, AB31940	1:100
Tau PTM (phosphorylation)	IF (S)	CP13 (Ser 202)	Kind gift from the Feinstein Institutes for Medical Research; developed by Peter Davies	1:200
Tau PTM (phosphorylation)	IF (S)	AT100 (Thr212, Ser 214)	Invitrogen, MN1060	1:500
Tau PTM (phosphorylation)	IF (S)	AT180 (Thr231)	Invitrogen, MN1040	1:500
Tau PTM (phosphorylation)	IF (S)	PHF1 (Ser396, Ser404)	Kind gift from the Feinstein Institutes for Medical Research; developed by Peter Davies	1:2000
Tau PTM (phosphorylation)	IF (S)	AT8	Invitrogen, MN1020	1:1200

*Note:* Details of methods and protocols are provided in Data S1.

Abbreviations: A, marker used in all cases; GFAP, glial fibrillary acidic protein; IF, immunofluorescence; IHC, brightfield immunohistochemistry; PTM, posttranslational modification; S, marker used in selected cases in pathology groups following AT8 analysis.

## 3 RESULTS

In the 12 pathology groups with refractory epilepsy overall 40% of cases were entirely negative, with low to moderate AT8 noted in the remainder, with some lesion‐specific patterns noted as detailed below.

### 3.1 FCD subtypes

**FIGURE 1:**
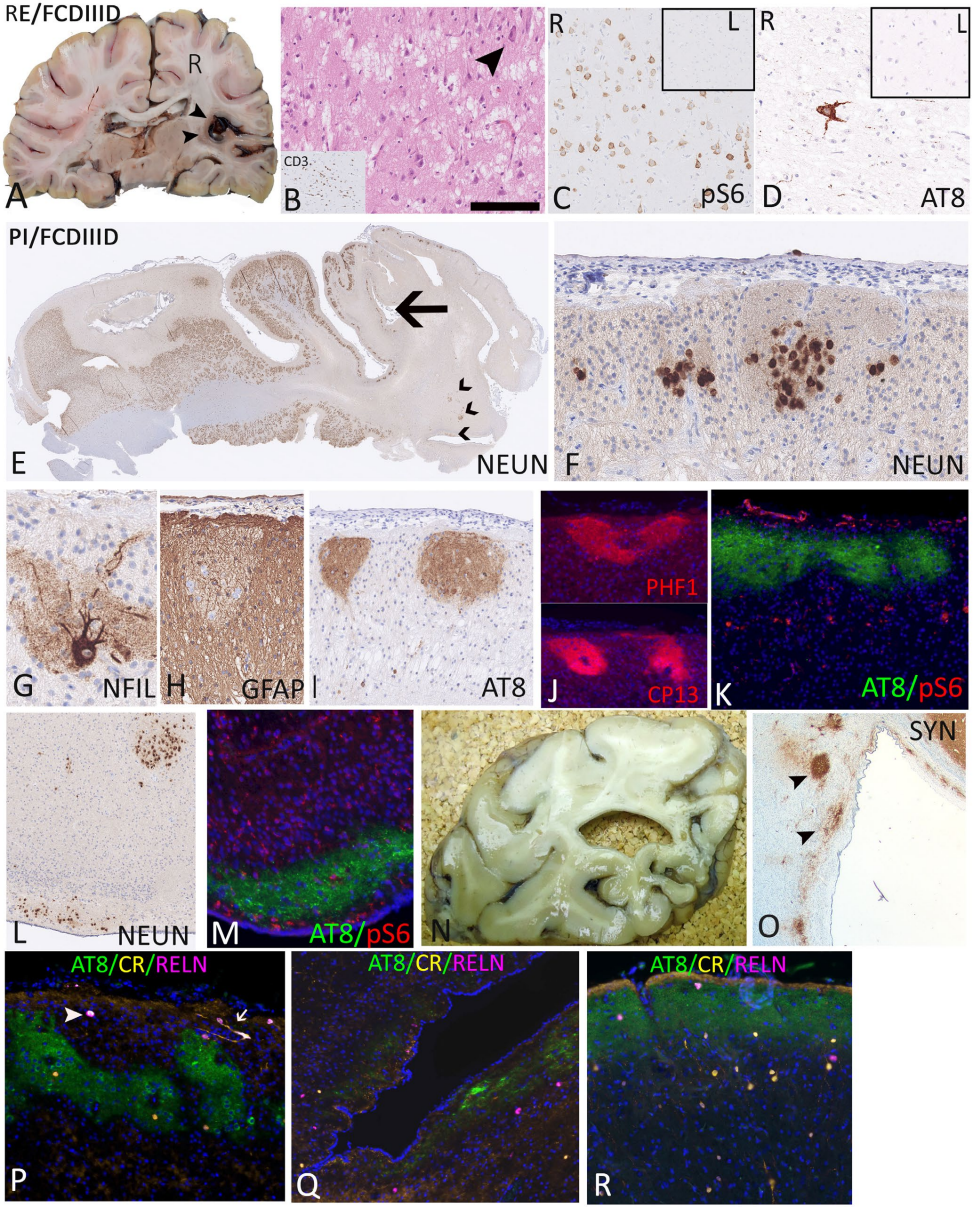
AT8 patterns in focal cortical dysplasia (FCD) type IIID. (A) FCD IIID/Rasmussen encephalitis (RE): a 37‐year‐old female with seizure onset at age 17 years, drug refractory with a clinical diagnosis on neuroimaging of RE and sudden unexpected death in epilepsy. Unilateral atrophy of the right periinsular cortex was confirmed, shown in a coronal slice of the fixed brain at the level of the lateral geniculate nucleus. (B) Histology‐confirmed unilateral cortical atrophy, vacuolation with scattered residual hypertrophic neurons (arrowhead), and CD3^+^ T‐cell nodules (inset), in keeping with active disease. (C) On the side of the inflammation, mTOR pathway upregulation was confirmed with numerous pS6‐positive neurons but absent in the contralateral insular cortex (inset). (D) AT8/pTau was increased on the damaged side in scattered neurons and neuropil threads with virtually absent staining in the contralateral hemisphere (inset). (E) FCD IIID/perinatal infarct (PI): a 25‐year‐old male with onset of epilepsy at age 12 years and a history of congenital heart disease corrected 1 day postnatally. Magnetic resonance imaging was consistent with a perinatal infarct in the occipital lobe, and resection sample with NeuN confirmed the typical microgyria, ulegyria (arrow), and cortical disorganization typical of FCD IIID/PI in addition to subcortical nodules of heterotopic neurons (chevrons). (F) In the microgyria region, residual neurons in layer I on NeuN stain, (G) scattered dysmorphic neurons on SMI32 neurofilament stain (NFIL), and (H) surrounding zones of dense gliosis were noted on glial fibrillary acidic protein (GFAP). (I) Immunopositivity in the residual islands of neurons and neuropil was shown with AT8 and also with (J) PHF1 and CP13. (K) pS6 upregulation was noted in small cells in the regions of tau deposition. (L) Periventricular regions showed islands of mature neurons on NeuN with focal AT8 and pS6 expression shown in panel M. (N) Postmortem sample from an 8‐year‐old subject with early onset epilepsy with a presumed perinatal infarct with typical ulegyria; (O) synaptophysin (SYN) immunohistochemistry confirmed islands of neurons at periventricular location. (P) AT8 multiplexed with calretinin (CR) and reelin (RELN) in the same case as panel E showed a reduction of CR neurons in the ulegyria region but pTau in both RELN^+^ (arrowhead) and CR^+^/RELN^+^ (arrow) positive neurons in the superficial cortex in the ulegyria region. (Q) CR^+^ and RELN^+^ small neurons were also noted in the periventricular nodules of neurons with AT8 expression. (R) Relative preservation and normal distribution of CR^+^ and RELN^+^ cells were noted in adjacent, better preserved cortex. Scale bar in B = 100 μm in B and D; 250 μm in C; 4 mm in E; 350 μm in F, H, I, J, K, L, M, P, Q, and R; and 150 μm in G. L, left; R, right.

We noted low levels of AT8 in FCD types IA, IIIA, and IIIB (Figure S1). In FCD IIID associated with Rasmussen encephalitis (RE), we included four surgical cases, three with chronic active RE showing "skip‐like" regions of cortical atrophy, active chronic inflammation, and scattered dysmorphic neurons (Figure S1D,E) and one with milder inflammation. In two postmortem (PM) cases of RE, tissue samples from involved and contralateral normal hemisphere were available for comparison. In one case (age = 57 years at death), "burnt out" RE was confirmed, with severe atrophy, minimal inflammation, and no significant AT8. In a second case (age = 37 years at death), focal right insular chronic active RE with scattered dysmorphic neurons in keeping with FCD IIID was observed (Figure 1A,B). pS6 showed labeling of the dysmorphic neurons in the FCD IIID region (Figure 1C) but was negative in the contralateral cortex (Figure 1C, inset). AT8 labeling in scattered neurons and threads, lateralized to the side of pathology (Figure 1D), whereas the contralateral hemisphere was negative (Figure 1D, inset).

In FCD IIID associated with perinatal infarct, we included six surgical cases and three PM cases with clinical, magnetic resonance imaging (MRI), and neuropathological features in keeping with perinatal ischemic infarcts, with microgyria and/or a "ulegyria" pattern with preferential atrophy at the sulcus (Figure 1E). Five of the cases involved parieto‐occipital regions (compatible with watershed region), and four were in the middle cerebral artery vascular territory. A history of birth injury following prolonged labor was documented in two cases, and in a further case there were cyanosis at birth and transposition of the great arteries, which was surgically corrected on day 1 of life. The remainder represented presumed perinatal ischemic strokes based on typical MRI and pathology features (Table S1 for case details).^21^ Disordered cortical lamination and atrophy with residual irregular islands of neurons separated by bands of myelin and gliosis, with scattered hypertrophic neurons highlighted with neurofilament immunohistochemistry, was confirmed in keeping with FCD IIID (Figures 1F–H and S2), involving ulegyric or microgyric cortex and with more normally laminated cortex often present at margins. In three cases (including the case with congenital heart disease), small nodules of neurons in the periventricular region and subcortical white matter were highlighted with NeuN and synaptophysin, in keeping with small heterotopia and representing a neuronal migration defect (Figure 1E,L,N,O,Q).

In two surgical cases, prominent AT8 in residual aggregates of neurons in layer II with diffuse neuropil/axonal labeling was observed (Figures 1I and S2) and in the periventricular heterotopia of one case (Figure 1M). Similar expression patterns with PHF1 and CP13 (Figure 1J) but less expression with AT100 and AT180 was noted with other pTau antibodies. Double labeling of AT8 with pS6 showed regional localization in the neuronal aggregates in FCD IIID and periventricular nodules (Figures 1K,M and S2), although cortical regions with pS6 expression and minimal pTau accumulation were also noted (Figure S7F). Neurodevelopmental markers reelin and calretinin showed a reduction in normal calretinin‐positive layer II neurons and Tbr1 in regions with AT8 and colocalization of AT8 in occasional calretinin^+^ and reelin^+^ neurons (Figure 1P,R). Calretinin‐ and reelin‐expressing cells were also present in periventricular regions, including areas with AT8 expression (Figure 1Q). In a further four perinatal infarcts, only rare AT8^+^ neurons, including in superficial cortical layer II neurons with comparable morphology to doublecortin‐positive cells, were noted (Figure S1F). In view of these findings in heterotopic neurons in FCD IIID, we included six gray matter heterotopia cases, which showed more variable AT8 labeling in four cases (Figure S3).

### 3.2 Vascular malformations

**FIGURE 2:**
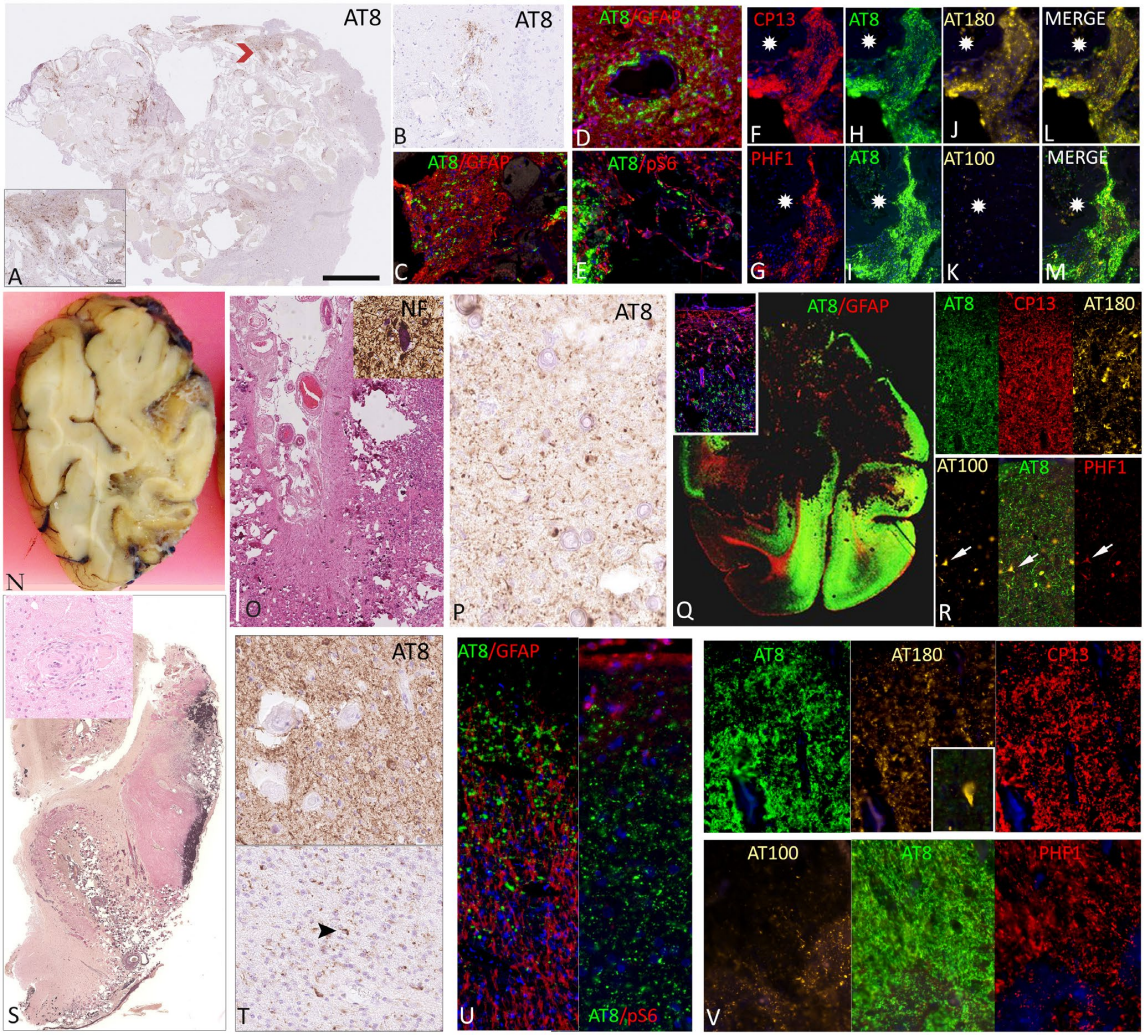
Phosphorylated tau in vascular lesions. (A–M) Cavernomas, (N–R) Sturge–Weber syndrome/leptomeningeal angiomatosis, and (S–V) meningioangiomatosis. (A) A male aged 56 years with duration of epilepsy of 5 years and a cavernoma (CAV) in the superior left temporal gyrus. AT8 immunohistochemistry showed positivity around the margins of the lesions and in parenchyma between vessels (region indicated with chevron shown in inset at higher magnification). (B) Cavernoma in a 67‐year‐old female with onset of epilepsy at age 61 years and a cavernoma near the hippocampus; AT8/pTau was in the perivascular region in the dentate gyrus, away from the main lesion. (C) A 62‐year‐old male with onset of epilepsy at age 36 years and a temporal cavernoma; double labeling with glial fibrillary acidic protein (GFAP) showed AT8‐pTau in regions of gliosis around the lesion and between vessels, but astroglial tau labeling was not defined. (D) A 42‐year‐old male with onset of epilepsy at age 31 years and a sclerosed cavernoma in the subiculum; perivascular AT8/pTau was not clearly associated with astroglial processes. (E) Same case as in panel C, with pS6‐234 showing intense labeling of endothelium and glia near the cavernoma in the region of AT8‐pS6 positivity. (F–M) pTau phosphorylation patterns in cavernoma (same case as in panel C); CP13 (F) showed equivalent labeling to AT8 (H) with less labeling for AT180 (J) and PHF1 (G) and lower expression of AT100 (K; asterisks indicate cavernoma intravascular space, the merged image of adjacent three markers is shown on the far right for each row [L, M]). (N) A 59‐year‐old patient with Sturge–Weber syndrome (SWS) and age at onset of epilepsy of 8 years with a resected right occipital lesion showing regions of cortical thinning and subcortical atrophy. (O) Hematoxylin and eosin (H&E) staining showing leptomeningeal angiomatosis and extensive vascularity and calcification in the superficial cortex (inset shows neurofilament [NF] SMI32 with scattered hypertrophic neurons). (P) AT8‐pTau showed intense labeling of threads, processes, and scattered neurons within the lesion. (Q) Lesional gliosis in AT8/GFAP section with inset showing perivascular and superficial cortex gliosis but no clear glial tau. (R) AT8 and CP13 showed more abundant staining in threads and processes than AT180, the later restricted to small cells and short, thick neurites. PHF1 and AT100 were also more restricted to small neurons (arrows) with less extensive threads compared to AT8. (S) Meningiomatosis (MA) in the temporal lobe in a 60‐year‐old female with onset of epilepsy at age 21 years, shown with elastic van gieson (EVG) to highlight the extensive region of cortical perivascular collagen (inset shows H&E staining with typical intracortical perivascular meningothelial cells). (T) AT8‐pTau (top) showed dense lesional cortical accumulation in neurons and threads with less in white matter (below) with occasional labeling of small glia cells (arrowhead). (U) A 34‐year‐old female with age at onset of seizures of 12 years; the lesion showed extensive gliosis, including superficial cortex layers, but no clear colocalization with AT8‐pTau; pS6 expression (right) was noted in layer I in regions with cortical AT8. (V) In the same case, tau phosphorylation patterns were similar to SWS, with dense AT8/CP13 but restricted AT180 expression to small neurons, although (inset) scattered AT180 neurons were striking in perilesional regions. PHF1 and AT100 were expressed in some processes but with overall less labeling than AT8. Scale bar in panel A = approximately 1 mm (inset bar in panel A = 250 μm); 500 μm in Q and N, and S; 50 μm in O; 80 μm in B and D; 100 μm in C; 120 μm in E–M, P, S inset, T, and V; and 250 μm in O, U, and R.

In 11 cavernomas, locations were cortical gray matter (*n* = 6), subcortical white matter, (*n* = 2) hippocampus (*n* = 2), or amygdala (*n* = 1), with one cortical lesion in the parietal lobe, one in the frontal lobe, and the remainder temporal. Focal AT8 accumulation was noted with all cavernomas except for the two located subcortically. Typical patterns were a rim of AT8 marginal labeling, coinciding with regions of microhemorrhage and gliosis, as threads, dense granular aggregates, and scattered cellular labeling (Figure 2A). AT8 was present in neuropil islands between back‐to‐back vascular channels (Figure 2A, inset). The density of pTau varied between lesions. In the cases with perilesional cortex or hippocampus, focal AT8 labeling was present in seven, including perivascular aggregates (Figure 2B) and prominent labeling of hippocampal dentate gyrus granular cells in one case. AT8/GFAP in three cases confirmed pTau in regions of dense fibrillary gliosis in the lesion but without distinct astroglial cell labeling (Figure 2C,D). AT8/pS6 in three cases showed pS6 expression in the lesion, including endothelial cells and glial cells, but no clear colocalization with pTau (Figures 2E and S7B). Other pTau epitopes showed comparable staining between CP13 and AT8 (Figure 2F,H) but lower levels of AT180 (Figure 2J) and PHF1 (Figure 2G); AT100 expression was much less abundant (Figure 2K). Labeling with pTau was also noted (particularly for CP13, AT8, and PHF1) in the marginal cortex, including the subpial layer (Figure S4D).

In Sturge–Weber syndrome (SWS; leptomeningeal angiomatosis), AT8 was present in eight of 10 cases, which showed typical pathology features (Figure 2N,O); in one case, tissue represented mainly marginal cortex. The density of AT8 did vary, however, from dense pan‐cortical deposits in threads and neurons (Figure 2P), including hypertrophic neurons, to predominant labeling of the superficial cortex in the region of calcification (Figure S4A) or cases with only rare grains or threads in the superficial cortex. Perivascular AT8 deposits were not striking in SWS, but labeling in interstitial white matter neurons was noted. AT8/glial fibrillary acidic protein (GFAP) showed extensive superficial cortical and perivascular gliosis but no significant astroglial tau (Figure 2Q and inset). AT8/pS6 showed regional colocalization but minimal cellular colocalization (Figure S7C). AT8 and CP13 showed more abundant staining in threads and processes in the lesion compared to AT180, which appeared restricted to coarser processes; PHF1 and AT100 were coexpressed in fewer intralesional neurons compared to more extensive AT8 in neurites throughout the neuropil (Figure 2R). AT8 labeling in the perilesional cortex was noted in five cases where tissue was available, including in the subpial layer and superficial cortical neurons.

Four cases of meningioangiomatosis showed typical pathological features (Figure 2S) and dense intralesional AT8 in neurites and neurons (Figure 2T, top), in keeping with previous observations.^22^ Labeling was noted to a lesser extent in perilesional cortex and white matter, with labeling of oligodendroglialike cells in one case (Figure 2T, bottom). AT8/GFAP, as in SWS cases, highlighted dense glial fibers and gliosis but no definitive astroglial tau (Figures 2U and S7A), and AT8/pS6 revealed pS6 mainly in layer I but minimal coexpression with AT8 (Figure 2U). CP13 labeling resembled AT8 in distribution, with reduced labeling of AT100, PHF1, and AT180 (Figure 2V). In the marginal cortex, however, there was an impression of higher and more specific AT180 labeling in pyramidal cells (Figure 2V, inset) compared to AT8 and CP13. In contrast, PHF1 and AT100 labeling was less expressed than AT8 in the marginal tissues.

### 3.3 Encephaloceles and scars

**FIGURE 3:**
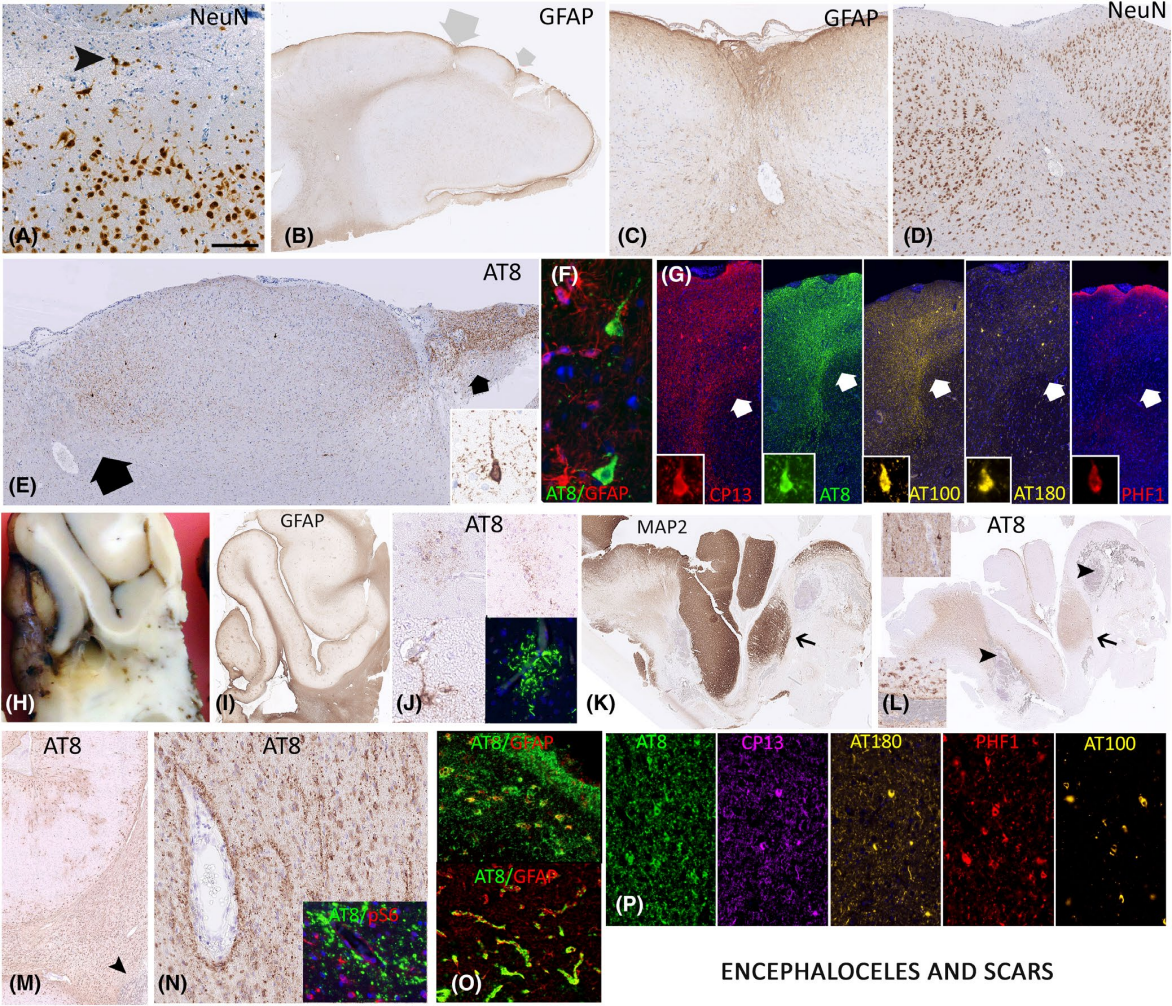
Tau pathology in encephaloceles and other cortical scars (A–G, encephaloceles, H–P, scars). (A) A 36‐year‐old male with a 6‐year duration of seizures; in the temporal pole, there was superficial gliosis and architectural abnormalities including excess neurons in layer I in keeping with a mild malformation of cortical development, and focal AT8 labeling was observed. (B) A 47‐year‐old female, with onset of epilepsy at 19 years and patchy superficial cortical scars on the inferior temporal gyrus mainly, shown on glial fibrillary acidic protein (GFAP; large scar with large arrow shown at higher magnification in panel C and NeuN showing loss of normal lamination in panel D). (E) AT8 showed aggregation of pTau at the sites of scarring, including neurons (inset) and threads. (F) There was no astroglia pTau noted on AT8/GFAP double labeling. (G) CP13 was equivalent to AT8 and AT100 labeling in the cortical scars, with less AT180 labeling and PHF1 restricted to a few superficial neurons only (arrows correspond to the scar indicated with a small arrow in panel B); insets confirm neuronal labeling in some neurons with all pTau epitopes (original magnification ×40). (H) A 54‐year‐old male with refractory epilepsy from the age of 9 years with a history of a road traffic accident at age 6 years with a traumatic brain injury, which was complicated by meningitis and an abscess, for which he underwent surgery. (I) This area shows a contusion on GFAP stain with cavitation and gliosis in the white matter. (J) AT8 showed astroglial tau in the cortex and perivascular deposits and granular aggregates. (K) A second resection sample corresponded to an area of cortical scarring, white matter gliosis, abundant macrophages, and presumed old surgical material (arrowheads in L and M). (K) Islands of preserved cortical gray matter were seen and (L) AT8 of same region showed abundant accumulation in white matter in astrocytes and in relation to operation cavity and macrophages with debris (arrowheads and lower inset) with islands of extensive cortical neuronal labeling (region near arrow, shown in upper inset at higher magnification). (M) Patchy cortical and extensive white matter tau is shown and (N) at higher magnification highlighting perivascular deposition; inset shows AT8 with pS6‐235, which showed strong expression of the latter in white matter glia and endothelium in regions of tau accumulation. (O) AT8/GFAP confirmed tau expression in Chaslin's subpial layer and layer I astroglia (top) and a relation between AT8 and perivascular glial foot process in the white matter (bottom). (P) Tau phosphorylation markers in the cortex showed variable labeling with lower expression of AT100 and AT180 compared to PHF1 and CP13. Scale bar in panel A = approximately 300 μm in A; 5 mm in B, H, I, K, and L; 250 μm in C,D, E, and G; 50 μm in F, J, and insets in E and G; 800 μm in M; and 200 μm in N, O, and P.

The pathology findings in the seven encephaloceles were variable; two cases showed small superficial defects on gyral crests of temporal lobe, highlighted with increased subpial GFAP labeling and a reduction of NeuN‐positive neurons (Figure 3B–D). In a further case in the temporal pole, increased layer I neurons with dyslamination was seen (Figure 3A), in keeping with a mild malformation but with the additional small linear cortical scars. A third case showed marked rarefaction and gliosis in the white matter but not in continuity with any overlying cortical defect. Remaining cases showed mild to moderate superficial cortical gliosis only. Two cases showed AT8 labeling localized to the regions with marked superficial gliosis (Figure 3E). In the remaining cases, AT8 showed scattered superficial cortical threads and grains and a subpial band (Figure S4B). AT8/GFAP did not confirm astroglial tau (Figures 3F and S4C) and pS6 minimal colocalization (Figure S7E). pTau epitopes showed lower levels of AT180, with PHF1 restricted to rare neurons in the scarred region (Figure 3G).

The etiologies of the cortical scars varied, including from prior traumatic brain injury (TBI), infection, and previous surgery (Table S1). Patchy and focal deposits of AT8 were observed in eight of 10 cases (Figure S5A–D). Significant pTau was present in only one case with childhood TBI complicated by cerebral abscess, which was operated on. In the region of a contusion (Figure 3H,I), subpial astroglial tau, granular astrocytes, and perivascular cortical AT8 labeling were suggestive of CTE but with no sulcal predilection (Figure 3J). Other regions, with sheets of macrophages and foreign material representing the operated abscess cavity, showed dense neuronal and glial AT8 with perivascular accumulation (Figure 3K–N). GFAP/AT8 confirmed coexpression (Figure 3O), and AT8/pS6 showed prominent pS6 in reactive glia in white matter and endothelium in regions of dense AT8 deposition (Figure 3N, inset), with the most prominent cellular colocalization in perilesional cortex (Figure S7D). pTau showed lower AT100 than AT8 and CP13, with intermediate PHF1 and AT180 expression in neurons and threads (Figure 3P). There was negligible AT8 in the small scars associated with prior electroencephalography (EEG; Figure S5).

### 3.4 Clinical correlations and quantitative analysis of tau

There were significant differences in the mean age of surgery (youngest in FCD IA, oldest in meningioangiomatosis), age at onset of epilepsy (youngest in FCD IA, oldest in cavernoma), and duration of epilepsy (shortest in FCD IA, longest in gray matter heterotopia) between pathology groups (Table 1). Episodes of recorded status epilepticus (more frequent in RE and heterotopia cases) were also different between groups but not for other seizure types, history of prior head injury, or prolonged febrile convulsions.

**FIGURE 4:**
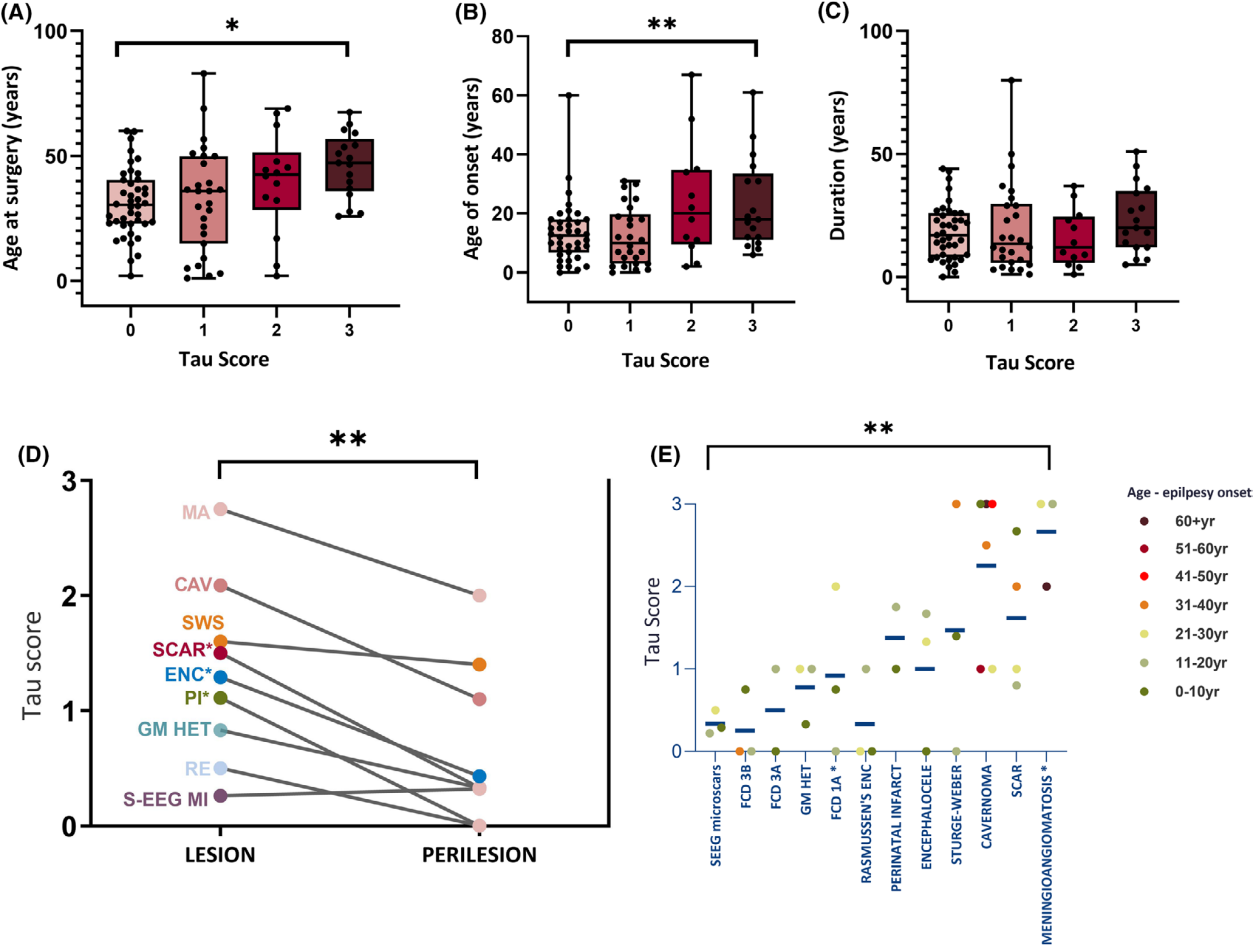
Analysis of tau scores in relation to age, underlying pathology, and perilesional tissue. (A, B) Lesional phosphorylated tau semiquantitative scores based on AT8 (see text for details of scoring scheme) were significantly higher in the whole series with (A) age at surgery (*p < .05) and (B) age at onset of epilepsy where information was available (**p < .005) but (C) not duration of epilepsy (linear regression analysis). The box plots represent mean and 25th to 75th percentiles; whiskers represent the maximum and minimum age, with all data points shown. (D) In the 74 cases where nonlesional adjacent tissue was available for comparison, lower tau scores were seen across all cases compared to the lesion (*p* < .001, Wilcoxon test) and within pathology groups were significantly lower for scars, encephaloceles, and perinatal infarcts (*p < .05). (E) Tau scores varied significantly across the 12 pathology groups (*p* = .003, chi‐squared test), with higher tau loads (blue bars indicate group mean) for vascular pathologies and scars. This scatter graph also groups cases according to age (decade of surgery), with dots representing mean tau score for the decade age group; higher tau loads with increasing age were noted in vascular lesions, although no significant regressions of tau with age were noted in any pathology group. CAV, cavernoma; ENC, encephaloceles; FCD, focal cortical dysplasia; GM, gray matter; HET, heterotopia; MA, meningiomatosis; MI, microinjury; PI, perinatal infarct; RE, Rasmussen encephalitis; SEEG, stereoelectroencephalography; SWS, Sturge–Weber syndrome.

AT8 was negative in the lesions in 42 of 104 cases. Beta‐amyloid in selected cases from pathology groups was negative (Table S1). The density of lesional pTau, on semiquantitative score, was greater with increasing age at surgery (linear regression, *p* = .02; Figure 4A). Age at onset of epilepsy was also greater in cases with greater lesional pTau (linear regression, *p* = .003; Figure 4B), but there was no significant relationship with duration of epilepsy (Figure 4C). AT8 was negative in the perilesional cortex in 50 of 74 cases, and the pTau score was significantly higher in the lesional than perilesional zone (*p* < .001, Wilcoxon test; Figure 4D).

There was a significant difference in mean pTau scores between pathology groups (*p* = .003, chi‐squared test), which was highest in vascular lesions and scars. Furthermore, within the pathology groups, there was a pattern of higher pTau scores with age at surgery, but as the groups were small, there was no significant age regression with any pathology type (Figure 4E).

There was no relationship between pTau scores and resected brain region; however, as there was a significant variation in resection site in relation to nature of underlying pathology (Table 1), this is a confounding factor. There was no relationship between tau pathology, seizure type (apart from a trend for higher tau with a history of status epilepticus, *p* = .05), gender, laterality, history of head injury, initial febrile convulsion, or seizure‐free outcome.

There was no difference in AT8 scores between surgical and PM tissue samples. There was a significant regression of AT8 score with year of surgery, which reflects the age of the paraffin tissue block (*p* < .01). Of note, however, there was no relationship between year of surgery and AT8 score within pathology groups, and furthermore the meningiomatosis group with the highest AT8 scores had the mean oldest tissue samples.

## 4 DISCUSSION

In a range of common epilepsy‐related acquired and developmental lesions, we report varied patterns of accumulation of pTau. pTau was often more associated with the lesion rather than widespread, with differences noted between pathologies that may reflect underlying drivers including seizures, mTOR activity, blood–brain barrier compromise, and localized brain injury. Importantly, pTau was absent in 40% of cases, suggesting individual vulnerabilities, and older patients were more susceptible in this cohort.

### 4.1 Epilepsy and tau phosphorylation

We confirmed common AD tau phosphorylation sites across epilepsy pathologies, with more consistent labeling with AT8 and CP13 compared to PHF1, AT100, and AT180, the later epitopes showing more variability (Figure S4); for example, PHF1 and AT180 were relatively low in SWS and cavernoma. Although there remains uncertainty regarding the most pathogenic molecular forms of tau,^3^ tau phosphorylation is widely considered a posttranslational modification (PTM) relevant to its aggregation.^23^ Although our pTau panel also represent physiological phosphorylation sites,^24^, ^25^ some sites, such as AT180, are known to enhance tau aggregation.^26^ In experimental models of TLE, pTau with AT100, PHF1, and CP13 was shown,^14^ whereas in status epilepticus a reduction of AT8 in the chronic stages and no changes with AT180 were noted.^27^ Reported surgical epilepsy series have not investigated tau PTMs in depth (see Table S2), and current findings may reflect different disease stages or lesion‐specific PTMs, as suggested in TS,^7^ which may be of biological relevance.

### 4.2 Localization of pTau and superficial cortex

There is considerable interest in the seeding of tau through brain networks in the progression of neurodegenerative diseases, which is enhanced by seizures.^28^ In our series, pTau load was greater in proximity to lesions than adjacent more normal‐appearing cortex, as also noted in previous studies of FCD II (Table S2). This raises the notion that some pTau accumulation in epilepsy may remain localized. pTau was previously reported in the subpial region of layer I in epilepsy,^1^ representing horizontal axons.^2^ Layer I axons include local and wider projections from cortical and subcortical inputs including the thalamus, considered relevant to cortical processing,^29^ with subpial transection used as a surgical procedure to reduce horizontal seizure propagation. Layer l axonal pTau may reflect abnormal network activity or possibly a transient response to surgery or anesthesia.^30^ Subpial tau also draws comparison to normal brain development, where tau phosphorylation remains elevated until the end of synaptogenesis, to enable microtubule plasticity during axonal growth.^31^ In fetal brains between 14 and 38 weeks, pTau‐Ser214 and to a lesser extent PHF1, CP13 with lower AT8 expression is limited to layer I, including cell aggregates.^17^ In epilepsy lesions, we noted layer I labeling often in perilesional regions with PHF1, CP13, and AT8 and a relative lack of labeling with AT180 and AT100, the later including phosphorylation sites at Ser 214, which therefore differs from late developmental patterns.^17^ Furthermore, we noted a potential vulnerability of superficial cortical layers to pTau across different pathologies including FCD I and III, SWS, and meningoceles. This draws comparison to tau distribution in Nodding syndrome, an endemic form of atonic epilepsy characterized by superficial cortical pTau,^32^ which differs from neuronal vulnerability patterns in AD.

### 4.3 pTau in relation to mTOR activation

Tau protein has diverse physiological functions beyond microtubule stability, many mediated through the mTOR pathway.^3^ In turn, mTOR activity indirectly regulates tau phosphorylation and degradation and importantly its autophagic clearance.^33^, ^34^, ^35^, ^36^ mTOR pathway activation occurs in neurons and glia in epilepsy surgical pathologies in addition to FCD II, as identified by downstream pS6 protein.^10^ In the current cases, although lesional and perilesional pS6 was confirmed, precise cellular coexpression with AT8 was often lacking (Figure S7). In one RE case, pTau lateralized to the side with marked pS6 neuronal upregulation, but pTau was not observed in other RE cases with active inflammation and mTOR activation. In summary, the role of mTOR in tau phosphorylation in epilepsy requires further experimental study, including at the single‐cell level.

### 4.4 Vascular dysfunction and pTau

pTau was more frequently present in vascular malformations in our series: 80% of SWS, 74% of cavernomas, and 100% of meningioangiomatosis. The association of pTau with meningioangiomatosis is long‐recognized, including in patients <40 years old.^22^ pTau accumulation in vascular malformations may relate to blood–brain barrier insufficiency, tissue hypoxia, oxidative stress, and glial dysfunction. mTOR signaling in cavernomas has been linked to its pathophysiology^37^; we noted pS6‐positive endothelium near pTau parenchymal deposits but minimal cell colocalization. Despite significant gliosis, we did not confirm astroglial tau in vascular malformations and found no beta‐amyloid. Impaired tau/pTau clearance also remains a potential mechanism, and there is increasing evidence for a role of an aging glymphatic system in tau accumulation^38^, ^39^ which may be relevant to observed pTau in vascular malformations.

### 4.5 Brain injury and pTau

Focal scars of diverse causes represent 4.9% of epileptogenic pathologies in surgical series,^19^ and we included scars following prior TBI and prior neurosurgery. However, significant neuronal and astroglia tau loads were observed in only one case, and there were no CTE‐like patterns. This contrasts with our previous PM series showing CTE in 15% of cases.^40^ Encephaloceles represent either congenital or acquired lesions, characterized by a protrusion of brain tissue through a defect in the skull^41^ and increasingly recognized in refractory TLE, representing between 1.9% and 12.5% of cases with reported underlying pathology of focal mild cortical malformation and/or cortical scarring and gliosis ^42^, ^43^, ^44^. We noted localized pTau accumulation in mainly superficial cortex in 29% of encephaloceles; this may relate to localized epileptogenic activity or injury. We have previously used microinjuries following electrodes implanted for the investigation of epilepsy to study cellular repair processes^45^, ^46^; the identification of rare nuclear pTau may be indicative of cellular stress during reorganization/repair.^47^ However, the predominant observation was absent pTau following electrode implantations across all stages of brain repair.

### 4.6 Developmental lesions

Compared to FCD II and TS,^7^, ^48^, ^49^ we noted limited pTau in FCD IA and acquired dysplasias FCD IIIA and IIIB and only focal pTau in malformation of cortical development and heterotopia, mainly in older cases. We did note distinct patterns of pTau in some perinatal infarcts associated with FCD IIID. Perinatal ischemic infarcts arise from the 20th gestational week to 28 days postnatal, and common causes including congenital heart disease (CHD) and neonatal asphyxia,^21^ but many remain unknown, representing "presumed perinatal ischaemic stroke" based on typical neuroimaging or pathology appearances.^50^, ^51^ The distinct cytoarchitectural changes in areas of damaged microgyric or ulegyric cortex have been argued to represent postnatal "acquired cortical dysplasias" with altered connectivity in remaining neurons,^52^ classified as FCD IIID.^18^ However, reduced brain weight and delayed gyrification on fetal MRI in CHD^53^ and reduced doublecortin^+^ neuroblasts, cortical growth, and NeuN/calretinin^+^ interneurons, but not NeuN/Tbr1^+^ neurons, in models of perinatal ischemia, support an additional developmental neuronal‐migratory defect.^54^ We noted prominent pTau in two perinatal infarcts and demonstrated reduced calretinin^+^ interneurons with AT8 colocalizing with calretinin/reelin^+^ neurons in the superficial cortex. Furthermore, lesional periventricular and white matter neuronal heterotopia were associated with pTau. In normal fetal development, tau/pTau is primarily in Tbr1^+^ neurons with lower expression in the geminal matrix^15^, ^17^ with the differential expression of tau isoforms and tau phosphorylation during development influencing neurophysiological functional maturity.^55^ Our findings warrant extended study of the relative vulnerability of neuronal subtypes to pTau in epilepsy pathologies of developmental or acquired causes. In addition, our observation of neuromigratory abnormalities challenges the concept that FCD IIID in perinatal infarcts represents acquired dysplasia arising after cortical migration is completed.^18^

Limitations of this study include that not every case had adjacent normal‐appearing cortex for comparison. As the pathology subgroups were relatively small, we did not include neuropsychology and cognitive testing for comparison with pTau accumulation, and we have no data for AD risk genes. We did not have precise information on localization of seizure activity, including from stereo‐EEG studies to compare with regional pTau. Finally, although no differences between PM and surgical samples were observed, we noted a relationship between AT8 scores and age of the tissue sample, which may have influenced immunohistochemistry as a further limitation.

In conclusion, in many epilepsy cases, there is minimal or absent pTau. Different patterns of pTau across epilepsy pathologies show an age‐related vulnerability but also expose multiple, alternative possible mechanisms at play. Further investigation is needed for understanding localized versus progressive and irreversible tau accumulation, PTMs, and specific neuronal vulnerabilities.

## AUTHOR CONTRIBUTIONS

*Immunohistochemistry protocols and lab preparations:* Alicja Mrzyglod, Anya Mebrouk, Hanaa El Hachami, Maritchka Ryniejska, and Joanna Bartkiewicz. *Clinical data, materials, and sample preparation:* Andrew McEvoy, Anna Miserocchi, Fenglai Xiao, Jane de Tisi, and Matthias Koepp. *Review of case material and pathology diagnosis:* Roland Coras, Ingmar Blumcke, and Maria Thom. *Study design:* Maria Thom, Joan Liu, and Matthias Koepp. *Write‐up, revisions, and review of manuscript:* all authors.

## FUNDING INFORMATION

This work is supported by the Wellcome Trust (Epilepsy and Neurodegeneration: Disease Mechanisms and Early Detection, 221 934/Z/20/Z). The Epilepsy Society supports the Epilepsy Society Brain and Tissue Bank at UCL Hanaa El Hachami and Maritchka Ryniejska, with additional equipment funding received from the Medical Research Council (reference MC PC_MR/X011860/1).

## CONFLICT OF INTEREST STATEMENT

None of the authors has any conflict of interest to disclose.

## ETHICS STATEMENT

The project has ethical approval (under UK HRA RES [S23/SC/0002]), and patients consented to the use of tissue for research. We confirm that we have read the Journal's position on issues involved in ethical publication and affirm that this report is consistent with those guidelines.

## Supporting information


Data S1.

Figure S1.

Table S1.

Table S2.

## Data Availability

All data are included in the article and Data S1.
