# Supplementary material for: Patterns of phosphorylated tau accumulation in a spectrum of acquired and developmental brain lesions associated with refractory epilepsy
Source: Epilepsia. 2025 Apr 29;66(8):3006–21. doi: 10.1111/epi.18418 (PMC12371652; doi:10.1111/epi.18418)
Supplement: Supplementary file 1 — Data S1. [file EPI-66-3006-s004.docx]

**Supplemental methods**

**Case selection detail**

104 cases were included, 83 cases from the Epilepsy Brain and Tissue bank (ESBTB) at University College London (UCL) with an additional 21 cases provided by the European Neuropathology Reference Center for Epilepsy Surgery in Erlangen (EpiCare). These included both surgical samples (n=93) and post-mortem tissues (n=11), obtained between 1991-2023, representing the following pathology diagnoses : **FCDIA** (n=11), **FCDIIIA** (FCD associated with HS, n=5), **FCDIIIB** (FCD associated with low grade glioneuronal tumours, n=6), **cavernomas** (with/without perilesional cortex) (n=11), **Sturge-Weber leptomeningeal angiomatosis** (SWS with/without FCDIIIC, n=10), **meningioangiomatosis** (n=4), **perinatal infarcts/ulegyria** (with FCD IIID, n=9), **Rasmussen’s encephalitis** (RE with FCD IIID, n=6), **grey matter laminar or nodular heterotopia** (n=6), **old scars** (including following previous surgery, trauma, abscess, n=10), **temporal lobe encephaloceles** (n=7). We also included cases with **focal micro-injuries** associated with previous intra-cranial stereo-EEG recordings as a comparison group (representing acute to chronic scars, n=19) ; the main epileptogenic pathology in this group represented : Hippocampal sclerosis (6), FCD II (7) mild malformation of cortical development (1), LEAT (1) and lesion negative (4). All the FCDIA cases and 8 SWS cases were provided by EpiCare with case groups summarized in Table 1 and further detail in supplemental Table 1. All patients had given specific consent for tissue to be used in research and the ESBTB has granted ethical approval for the project.

**Staining panels overview**

From all 104 cases, further 5 micron FFPE sections were selected to represent the maximal extent of the lesion with adjacent normal-appearing perilesional cortex if available (or from a second tissue block), and immunolabelled using AT8 (pTau Ser202/Thr205, ThermoFisher Scientific MN1020, 1:1200) ),a tau phosphorylation site routinely used in evaluation of the stages primary and secondary tauopathies, and scanned at x40 (Hamamatsu 360, Hamamatsu photonics, Nanozoomer S360). In cases showing a higher amount of AT8 labelling (See supplemental Table 1), further immunohistochemistry was carried out for Amyloid Beta (Dako, M0872, 1:50,) and multiplex labelling of AT8 with mTOR pathway activation marker (pS6 235-236 ,Cell signalling technology, #4857, 1:200), GFAP for astroglial tau (Dako, 1:2500), Calretinin (Merck, anti-rabbit, C7479, 1:100 ), reelin (Merck, anti-mouse,MAB5366, 1:500), Tbr1 (Abcam, anti-rabbit, AB31940, 1:100,) as well as for other Tau phosphorylation sites (PHF1, Ser396/Ser404, gift from Feinstein Institutes 1:2000; AT100, Thr212/Ser214, Invitrogen MN1060, 1:500; AT180, Thr23 Invitrogen MN1040, 1:500; CP13, Ser202, Gift from Feinstein Institute, 1: 200) using a Ventana Discovery immunostaining platform (Supplemental methods for detail). Immunofluorescence sections were scanned with a Hamamatsu NanoZoomer S60 Digital slide scanner at x40 (C13210-04, Hamamatsu Photonics).

**Immunostaining protocols**

**AT8 labelling**

Sections were cut at five microns. Immunohistochemistry was carried out in all cases for AT8 (pTau Ser202/Thr205, Monoclonal Antibody, ThermoFisher Scientific MN1020, 1:1200) with the Discovery Ultra autostainer (Ventana, Roche). IHC staining was performed on a Discovery Ultra autostainer (Ventana, Roche). The slides were loaded on the trays, deparaffinized before antigen retrieval with CC2 (citrate buffer, pH 6). Endogenous horseradish peroxidase activity was quenched with Inhibitor CM followed by the addition of primary antibody anti-AT8 (ThermoFisher Scientific, 1:1200). The primary antibody was amplified with the Amplification kit (05266114001, Roche) and anti-mouse HRP secondary antibody (05269652001, OmniMap Anti-Mouse HRP, Roche). 3,3'-Diaminobenzidine 28 (DAB) was applied for visualization of the epitope (ChromoMap DAB, 05266645001, Roche) followed by a haematoxylin counterstain for visualisation of cellular nuclei (Hematoxylin II, 05277965001). The slides were then dehydrated with graded alcohols, xylene and coverlipped in another autostainer (HistoCore, SPECTRA ST, Leica). All sections were scanned by a digital slide scanner at 40x magnification(Hamamatsu 360, Hamamatsu photonics, Nanozoomer S360).

**Semi Quantitative method for AT8**

AT8-labelled sections were qualitatively evaluated by three observers for the presence of any cellular labelling, patterns and distribution (AMe, AMr, MT). In addition a semi-quantitative score was devised for the evaluation of tau pathology associated with lesional area as well as a separate score for perilesional tissue: Score 0 for completely negative, 1 for rare (<5), 2 for few (5-50) and 3 for abundant AT8+ neurites and/or neuronal or glial based on the region with maximal staining in the tissue section.

**pS6 labelling**

For the demonstration of pS6, two antibodies recognising different phosphorylation sites of pS6 were used: ser240/244 specific for mTORC1 pathway, and ser 235/236 which is a phosphorylation site that may be mTOR-independent through Ras-MAPK pathway. For manual staining, 5 μm thickness formalin-fixed, paraffin-embedded brain sections of each case were processed through xylene and graded alcohols before immersion in a solution with 0.9% hydrogen peroxide for 15 minutes. Sections were microwaved in unmasking buffer (H-3301; Vector Laboratories Inc., USA) at full power for 12 minutes, and cooled for 20 minutes. Sections were blocked using 2.5% normal horse serum (Vector Lab, Peterborough, UK) for 20 minutes before incubation in a solution containing anti-phospho-S6 ser240/244 (1:1000, #5364, Cell Signaling Technology, Inc., Danvers, MA, USA) or ser235/236 (1:150, #4857, Cell Signaling Technology, Inc.) overnight at 4ºC. DAKO REAL Envision horseradish peroxidase (HRP) solution (DAKO, Cambridgeshire, UK) was applied for 30 minutes and diaminobenzidene chromogenic activation was performed.

**Beta amyloid labelling**

The slides were subjected to a deparaffinization process using xylene and manually rehydrated with graded alcohol. Subsequently, the slides were treated with formic acid for 15 minutes, followed by a 5-10 minute rinse in running tap water. Finally, the slides were loaded into the Discovery Ultra autostainer from Ventana (Roche) where antigen retrieval using Ultra CC2 was completed. Any endogenous peroxidase activity was quenched by Inhibitor CM followed by hand application of 100 μL addition of the primary antibody Beta-Amyloid (Dako, 1:50, M0872). DAB CM was applied for visualization followed by a Haematoxylin II counterstain for visualisation of cellular nuclei. The slides were then placed in an automated cover slipper. All sections were scanned by a digital slide scanner (Hamamatsu 360, Hamamatsu photonics, Nanozoomer S360).

**Multiplex labelling**

This was conducted on representative pathologies (detailed in Supplemental Table 1)

**GFAP and AT8**

Double IHC staining of GFAP and AT8 was performed by the Discovery Ultra autostainer (Ventana, Roche). The slides were loaded on the trays, deparaffinized and pretreated before antigen retrieval with Protease-1 (05266688001, Roche). The rabbit polyclonal primary antibody anti-GFAP was added (Dako, 1:2500) followed by Red 610 kit for its visualization (07988176001, Roche). The primary antibody and HRP links from the previous staining sequence were then denatured with CC2 buffer at 100^o^C for 8min then staining protocol for the second primary antibody anti-AT8 (ThermoFisher Scientific, 1:1200) was performed with citrate buffer retrieval at 91 ^o^C for 24min (CC2, Roche, 05424542001). FAM kit (07988150001, Roche) was then used for visualization of AT8 followed by the counterstain DAPI to visualize cellular nuclei (QD DAPI, 05268826001, Roche). All sections were scanned by the Hamamatsu NanoZoomer S60 digital scanner (Hamamatsu, Hamamatsu photonics) with Alexa 594, FITC and DAPI filters.

**Double labelling with pS6 and AT8**

Double IHC staining of PS6 and AT8 was performed by the Discovery Ultra autostainer (Ventana, Roche). Antigen retrieval was performed with citrate buffer CC2 at 91 ^o^C (CC2, Roche, 05424542001). The primary antibody anti-pS6 (ser235/236) and (Ser240/244) was added (Cell signaling technology, 1:200, #4857) followed by Red 610 kit for its visualization. One denaturation cycle was used as in the above protocol and staining for AT8 was performed (ThermoFisher Scientific, 1:1200). FAM was then added for visualization of AT8 followed by the counterstain DAPI to visualize cellular nuclei. Again, all slides were scanned with the Hamamatsu NanoZoomer S60 digital scanner.

**QuPath quantitative analysis**

In the selected cases studied from different pathology groups (Supplemental Table 1), QuPath software (Bankhead, Loughrey et al. 2017) was used to quantify the labelling index of AT8 and pS6-240 in a region of interest (ROI) near the lesion with maximal pTau labelling and a comparable size ROI in remote perilesional cortex. The percentage of co-localisation (overlap) of AT8 with pS6 was calculated and also the overlap of pS6-240 with AT8 using a QuPath script (developed and available on request from AMr) (Supplemental Figure 7).

**Immunofluorescence method for pTau epitopes**

| **Tau and other antibodies** | **Source, Clone** | **Clonality** | **Method** | **Dilution** | **Pre-treatments, details** |
| --- | --- | --- | --- | --- | --- |
| CP13 (Ser 202) | Kind gift from the Feinstein Institutes for Medical Research; developed by Peter Davies | Mouse, Monoclonal | Ventana Discovery autostainer (Roche) | 1:200 | Detection performed with Cy5 kit (  07551215001); epitope retrieval: CC1, 24min, 91^o^C  (06414575001) |
| AT100 (Thr212, Ser 214) | MN1060, Invitrogen | Mouse, Monoclonal | Ventana Discovery autostainer (Roche) | 1:500 | Detection performed with Rhodamine 6G kit (  07988168001); epitope retrieval: CC1, 24min, 91^o^C  (06414575001) |
| AT180 (Thr231) | MN1040, Invitrogen | Mouse, Monoclonal | Ventana Discovery autostainer (Roche) | 1:500 | Detection performed with Rhodamine 6G kit (  07988168001); epitope retrieval: CC1, 24min, 91^o^C  (06414575001) |
| PHF1 (Ser396, Ser404) | Kind gift from the Feinstein Institutes for Medical Research; developed by Peter Davies | Mouse, Monoclonal | Ventana Discovery autostainer (Roche) | 1:2000 | Detection performed with Cy5 kit (  07551215001) epitope retrieval: ULTRA CC2, 24min, 91^o^C  (05424542001) |
| AT8 (IF) | MN1020, Invitrogen, 1:1200, | Mouse, Monoclonal | Ventana Discovery autostainer (Roche) | 1:1200 | Detection performed with FAM kit (  07988150001) epitope retrieval: ULTRA CC2, 24min, 91^o^C  (  05424542001) |

Single immunofluorescence labelling protocols were developed for each of the five antibodies against tau phosphorylated isoforms. The optimum epitope retrieval conditions and primary antibody concentrations were established per marker as follows: AT8 (ULTRA CC2, 24min, 91^o^C; 1:1200), AT100 (CC1, 24min, 91^o^C; 1:500), AT180 (CC1, 24min, 91^o^C; 1:500), CP13 (CC1, 24min, 91^o^C; 1:200) and PHF1 (ULTRA CC2, 24min, 91^o^C; 1:2000). The epitopes were then labelled with one of the following tyramide-amplified fluorophores: Cy5 (07551215001, Roche), Rhodamine 6G (07988168001, Roche) or FAM (07988150001, Roche) and counterstained with DAPI nuclear marker (QD DAPI, 05268826001, Roche). The stained slides were scanned with S60 Hamamatsu Fluorescence Whole Slide scanner with the following fluorescence filters: Alexa 568, Alexa 647, FITC and DAPI with the most optimal exposures per fluorophore.

Bankhead, P., et al. (2017). "QuPath: Open source software for digital pathology image analysis." Sci Rep **7**(1): 16878.

QuPath is new bioimage analysis software designed to meet the growing need for a user-friendly, extensible, open-source solution for digital pathology and whole slide image analysis. In addition to offering a comprehensive panel of tumor identification and high-throughput biomarker evaluation tools, QuPath provides researchers with powerful batch-processing and scripting functionality, and an extensible platform with which to develop and share new algorithms to analyze complex tissue images. Furthermore, QuPath's flexible design makes it suitable for a wide range of additional image analysis applications across biomedical research.
