# Supplementary material for: Patterns of phosphorylated tau accumulation in a spectrum of acquired and developmental brain lesions associated with refractory epilepsy
Source: Epilepsia. 2025 Apr 29;66(8):3006–21. doi: 10.1111/epi.18418 (PMC12371652; doi:10.1111/epi.18418)
Supplement: Supplementary file 2 — Figure S1. [file EPI-66-3006-s003.zip › epi18418-sup-0007-Supplemental Figures Legends Revision.docx]

**Supplemental Figures**

**Supplemental Figure 1. Observations of AT8 in FCD subtypes**

**Summary of group findings**

(A) FCDIA: In eight of eleven cases with lesions in the occipital lobe, only rare threads or grains were noted, mainly in layer II with occasional neurones showing short radial processes. There was no glial, subpial, white matter or periventricular labelling. (B) FCDIIIA: These cases showed typical pathology with extensive neuronal loss in temporal neocortical layer II/III and clustering of neurones in layer II. In two of five cases scattered threads and grains were noted in the superficial cortex with rare neuronal labelling ; the sclerotic hippocampus was not included in the analysis.(C) FCDIIIB: In two of six cases, AT8 labelling was seen in peri-tumoral dyslaminar cortex as rare neurones or threads in layer II, including one case with previous surgery. Increased AT8 was variably present, in threads, axons and dysmorphic ganglion and glial cells associated with glioneuronal tumours (Inset in C). The increased AT8 in the glioneuronal tumoral component was not included in the quantitative evaluation.(D). Rasmussen’s encephalitis case with areas of cortical collapse and dysmorphic neurons (E), consistent with features of FCD Type 3D were studied; surgical cases were negative for AT8 (not shown). (F). In many perinatal infarcts with FCD 3D only rare AT8 neurones in layer II, similar to doublecortin (DCX) positive neurones, were noted.

Bar in A, B, F equivalent to approximately 50 µm, C approximately 25 µm, D approximately 50 µm, E approximately 100 µm.

**Supplemental Figure 2. AT8 patterns in a perinatal infarct**

(A) A 27-year-old male with refractory epilepsy and an occipital perinatal infarct of unknown cause with the typical features of FCD IIID confirmed on NeuN with nodular aggregates of cortical neurons in the FCD IIID region. (B) GFAP from the region arrowed in A show dense glial processes in region of ulegyria around the neurons. (C) pS6-235 for mTOR pathway activation in same region as (A) shows labelling in scarred cortex mainly in cells with glial morphology showed at higher magnification (same region as B) in (D) ; inset pS6-240 in same area. (E) AT8 (in same region as A&C) shows labelling in residual nodules of gray matter in superficial cortex with diffuse axonal pattern, shown in (F) at higher magnification (same region as B&D) and inset in small cells in layer 2.

Bar is equivalent to approximately 1mm in A,C, E and 250 microns in B,D,F.

**Supplemental Figure 3. AT8 in grey matter heterotopia** Six post-mortem cases with subcortical band heterotopia (2) and periventricular nodular heterotopia (4), included two cases as part of a complex malformation with overlying polymicrogyria or cortical dysplasia.

AT8 expression was noted in four but more prominent in one case with scattered neurones in subependymal nodules, periventricular glia and overlying cortex with polymicrogyria, with pTau in superficial cortical layer II neurones and subpial astrocytes in this region (3A-C). This was a 69-year-old female with a long history of seizures and a complex malformation with bilateral periventricular heterotopia in the temporal horn (arrowhead) and overlying cortical malformation with focal polymicrogyria (arrow) (LFB/CV section) (B). Abnormal cortex near arrow showed moderate AT8 labeling, greater than in normal cortex (not shown) and (C) scattered AT8-positive neurones and more abundant AT8-positive subependymal glia were present in the heterotopia. In three further cases, scattered AT8+ neurones in the heterotopia and layer II neurones in the overlying cortex were noted with focal subpial astroglia: (D). A 69-year-old female with onset of seizures age 19 and parietal laminar heterotopia identified at post-mortem (arrowhead) (E). Occasional AT8- positive neurones were observed in both the heterotopia and normal cortex. (F). 51-year-old female with onset of epilepsy age 22 and a diagnosis of periventricular nodular heterotopia; there were infrequent AT8+ neurites in the heterotopia and overlying cortex. Bar shown in C for B, E and F approximately 250 µm.

**Supplemental Figure 4. Subpial pTau and superficial cortex labelling.**

(A). SWS in a 44-year-old patient at the time of surgery showing a gradient of labelling from the superficial to deep cortex in the lesion with more AT8 in layer II compared to layer III and IV. (B). SWS in a one-year-old patient with moderate intensity subpial band of AT8 axonal labelling in the perilesional cortex. (C). Encephalocele with subpial AT8 but no colocalization with GFAP highlighting the subpial Chaslins’ gliosis. (D). Cortex adjacent to a cavernoma showing subpial pTau was also prominent with PHF1 less so with AT180 and AT100 (not shown).

**Supplemental Figure 5.**

**Patterns of pTau in scars**

(A). A 17-year-old with refractory epilepsy following an episode of childhood meningitis complicated by cortical scarring and hippocampal sclerosis. pS6-240 and inset pS6-235 both showed regional labelling of glial cells and neurons in the damaged microgyric cortex. (B) AT8 showed very focal labelling in superficial damaged cortex, layer I arrows shown in higher magnification in (C) and scattered residual neurons were AT8 positive (D, arrow).

**pTau in Intracranial S-EEG microinjuries**

Electrode track scars (cortical/white matter) or surface subdural-grid micro-injuries in 21 cases, of age 5 to 329 days (interval from implant to surgical resection), represented acute to chronic small injuries as previously described ^1^. Minimal AT8 was observed, with some nuclear AT8 expression in neuronal and glial cells in relation to these lesions and mainly in more acute injuries. This contrasted with the more extensive pS6-expression in glial, endothelial and neuronal cells at the lesion site and as previously described ^2^. These findings support that brain injury and normal stages of reorganization and repair are not accompanied by pTau accumulation. We do not have information on the EEG activity at the specific track scars studied. We have previously used microinjuries as a model for cellular repair ^1,3^ and the identification of some nuclear pTau may be indicative of cellular stress in this context ^4^.

(E, F) A 10 day old lesion following electrode implantation and tissue resection with the core of the scar cavity showing pS6 upregulation with expression observed in glia and endothelial cells in addition to neurons at the organizing margin (shown in G ; arrow indicates endothelial cells). (H) There was infrequent AT8 labelling in these lesions from acute stages (10 days or less), to subacute (11-69 days) to chronic (70 days +) post implantation, but with an impression of increased neuronal nuclear (H, I) and cytoplasmic AT8 in subacute lesions but (J) this was not statistically significant. A, B x 1.2, C,D,E,F x 10, G x 20 and H,I x 64 original magnifications.

**Supplemental Figure 6. pTau phosphorylation comparisons between pathologies.**

Heat map for the qualitative immunostaining of pTau epitopes in epilepsy lesional pathologies as assessed on multiplex labelling. Focal epilepsy lesions share similar relative abundance of CP13 and AT8 Sturge-Weber and meningio-angiomatosis both show similar patterns with less AT100, AT18 and PHF1 compared to AT8/CP13. Perinatal infarcts show relatively higher PHF1 than other pathologies.

**Supplemental Figure 7. Quantitative analysis of Ps6-240 and AT8 in pathology groups**

For all cases pS6-240, AT8 and merged image is shown in both lesional region of interest (ROI) (left panel) and the perilesional grey matter ROI in adjacent cortex (right panel); all shown at x 40 magnification. Using Qupath (see supplementary methods) the labelling index (LI) for each marker is shown for whole ROI (not just the field shown) and indicated as a percentage (top left each figure). In the merged images the percentage of pixel overlap (i.e. co-localization) of AT8 with pS6 is shown in green text and the percentage of overall of pS6 with AT8 is shown in red text.

(A) Meningioangiomatosis, showing high AT8 LI in the lesion and higher co-localization fraction with pS6 than adjacent cortex. (B) Cavernoma with low co-localization fractions. (C) SWS with higher AT8 LI in lesion than perilesional cortex but low pS6 LI in both regions. (D) Scar with uniformly higher AT8 LI in ROI but higher co-localization with pS6 in perilesional cortex. (E) Encephalocele with focus of higher cortical AT8 LI but low co-localization fractions with pS6. (F) With higher AT8 LI in lesional tissue but greater co-localization fraction with pS6 in the marginal better preserved cortical ROI.

1. Goc J, Liu JY, Sisodiya SM, Thom M. A spatiotemporal study of gliosis in relation to depth electrode tracks in drug-resistant epilepsy. Eur J Neurosci 2014;39(12):2151-62. DOI: 10.1111/ejn.12548.

2. Liu J, Reeves C, Michalak Z, et al. Evidence for mTOR pathway activation in a spectrum of epilepsy-associated pathologies. Acta Neuropathol Commun 2014;2:71. DOI: 10.1186/2051-5960-2-71.

3. Reeves C, Pradim-Jardim A, Sisodiya SM, Thom M, Liu JYW. Spatiotemporal dynamics of PDGFRbeta expression in pericytes and glial scar formation in penetrating brain injuries in adults. Neuropathol Appl Neurobiol 2019;45(6):609-627. DOI: 10.1111/nan.12539.

4. Younas N, Saleem T, Younas A, Zerr I. Nuclear face of Tau: an inside player in neurodegeneration. Acta Neuropathol Commun 2023;11(1):196. DOI: 10.1186/s40478-023-01702-x.
