# Supplementary figures and images for: Patterns of phosphorylated tau accumulation in a spectrum of acquired and developmental brain lesions associated with refractory epilepsy
Source: Epilepsia. 2025 Apr 29;66(8):3006–21. doi: 10.1111/epi.18418 (PMC12371652; doi:10.1111/epi.18418)

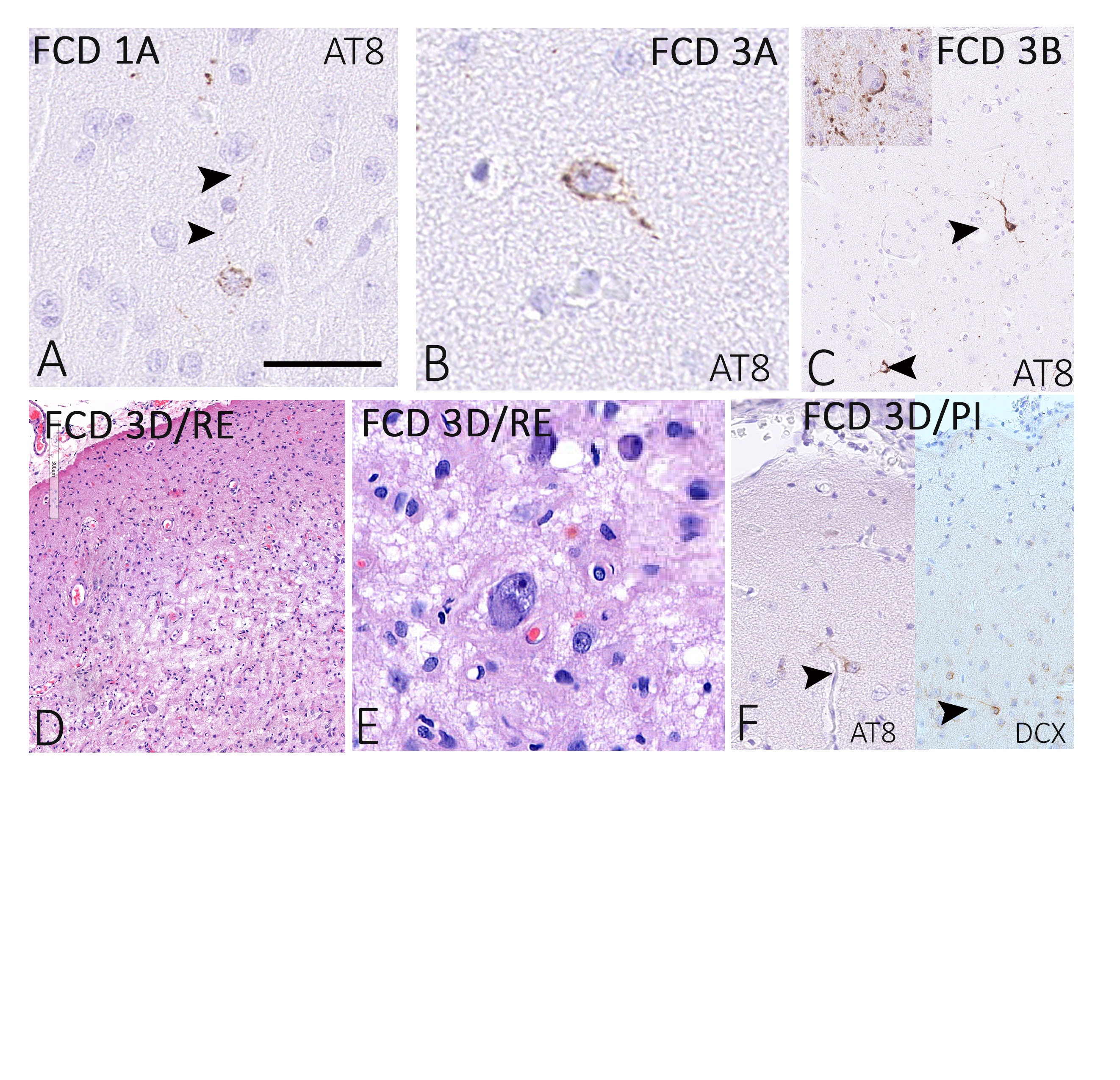

Supplement: Supplementary file 2 — Figure S1. [file EPI-66-3006-s003.zip › epi18418-sup-0001-supinfo_Supplemental figure 1.jpg]

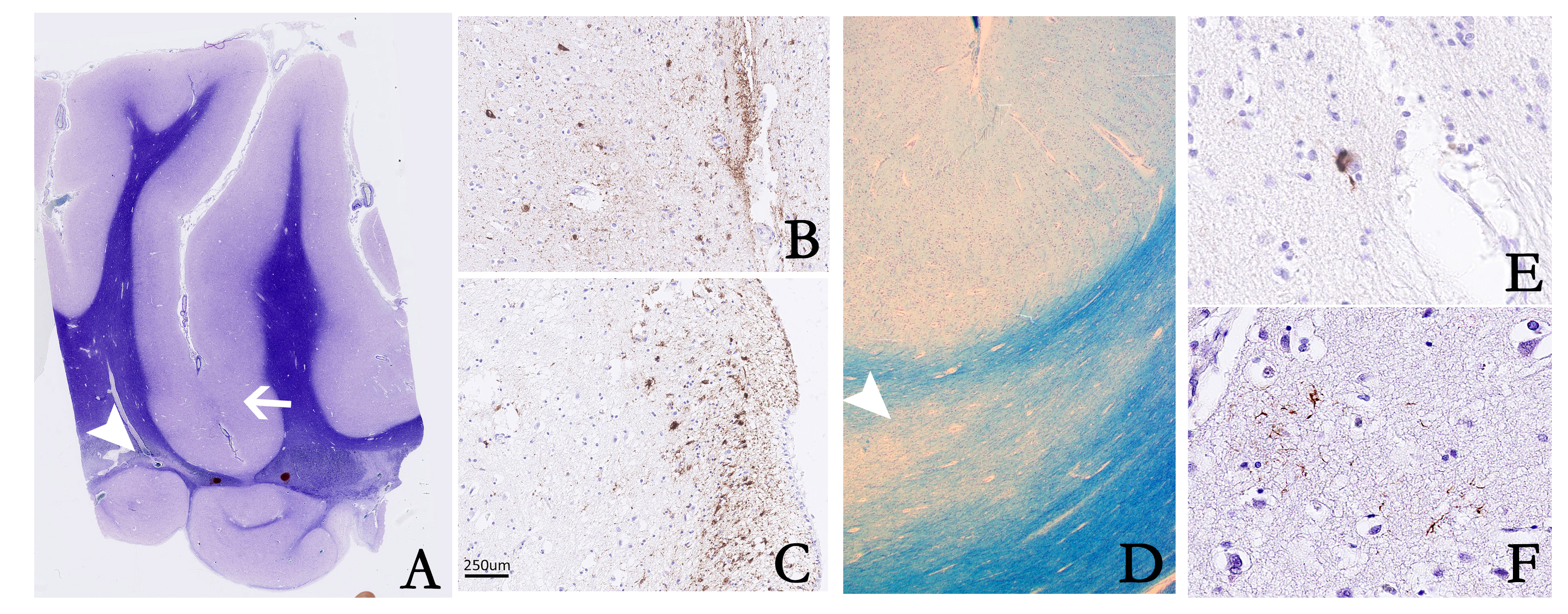

Supplement: Supplementary file 2 — Figure S1. [file EPI-66-3006-s003.zip › epi18418-sup-0002-supinfo_Supplemental Figure 3.jpg]

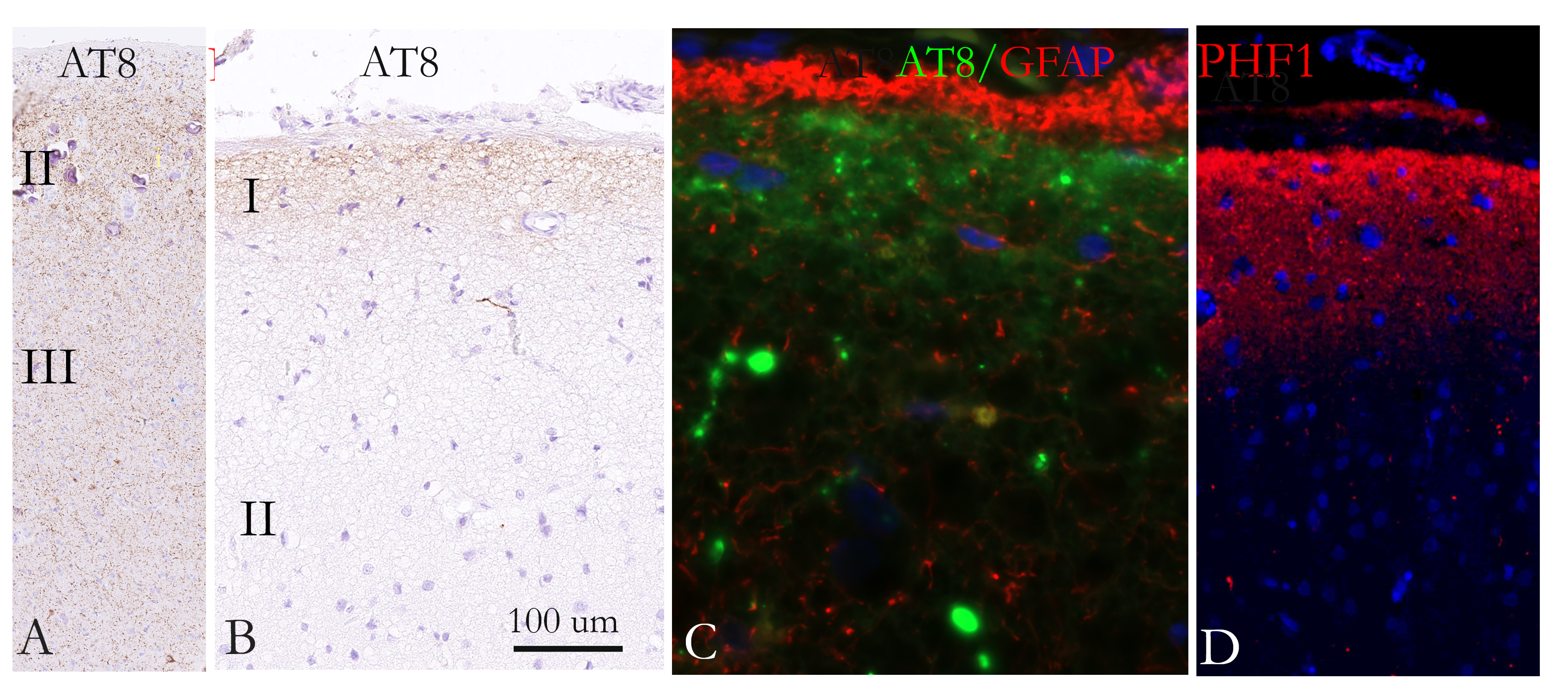

Supplement: Supplementary file 2 — Figure S1. [file EPI-66-3006-s003.zip › epi18418-sup-0003-supinfo_Supplemental figure 4. copy.tif]

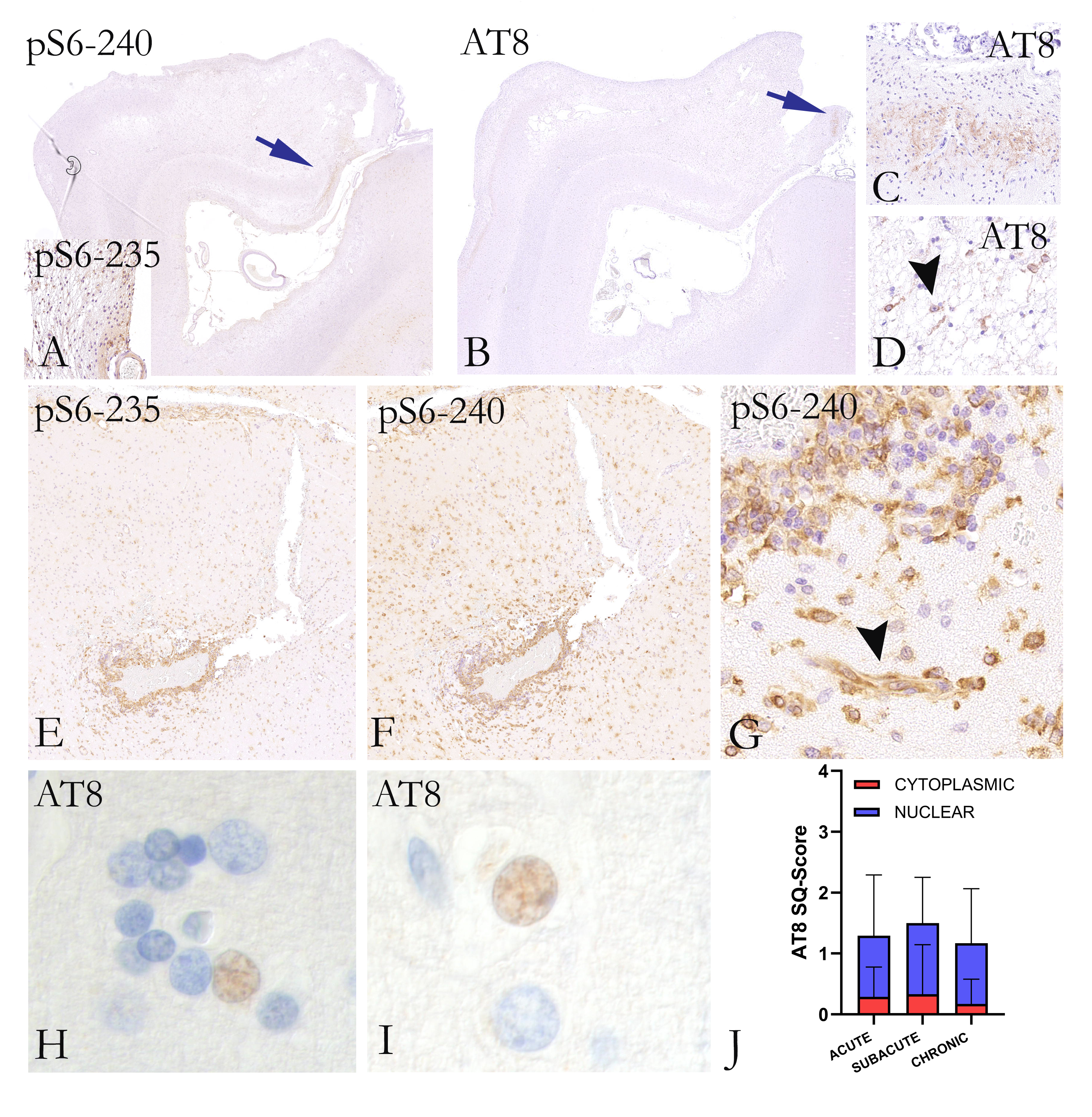

Supplement: Supplementary file 2 — Figure S1. [file EPI-66-3006-s003.zip › epi18418-sup-0004-supinfo_Supplemental Figure 5 copy.tif]

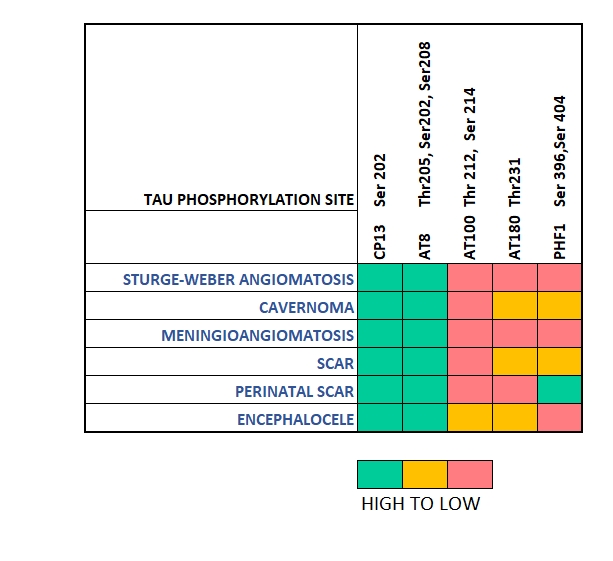

Supplement: Supplementary file 2 — Figure S1. [file EPI-66-3006-s003.zip › epi18418-sup-0005-supinfo_Supplemental figure 6.jpg]

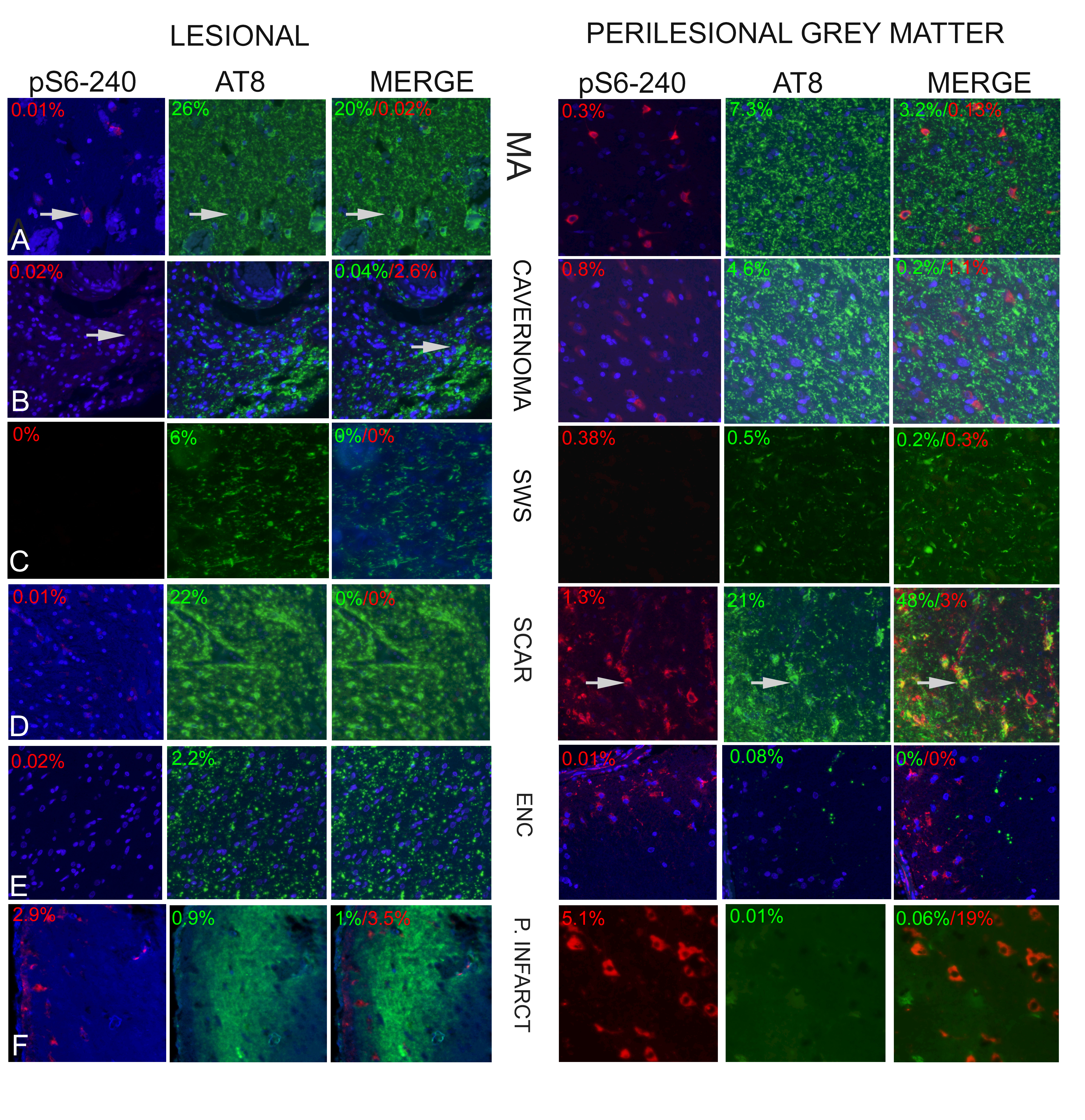

Supplement: Supplementary file 2 — Figure S1. [file EPI-66-3006-s003.zip › epi18418-sup-0006-supinfo_Supplemental Figure 7 copy.tif]

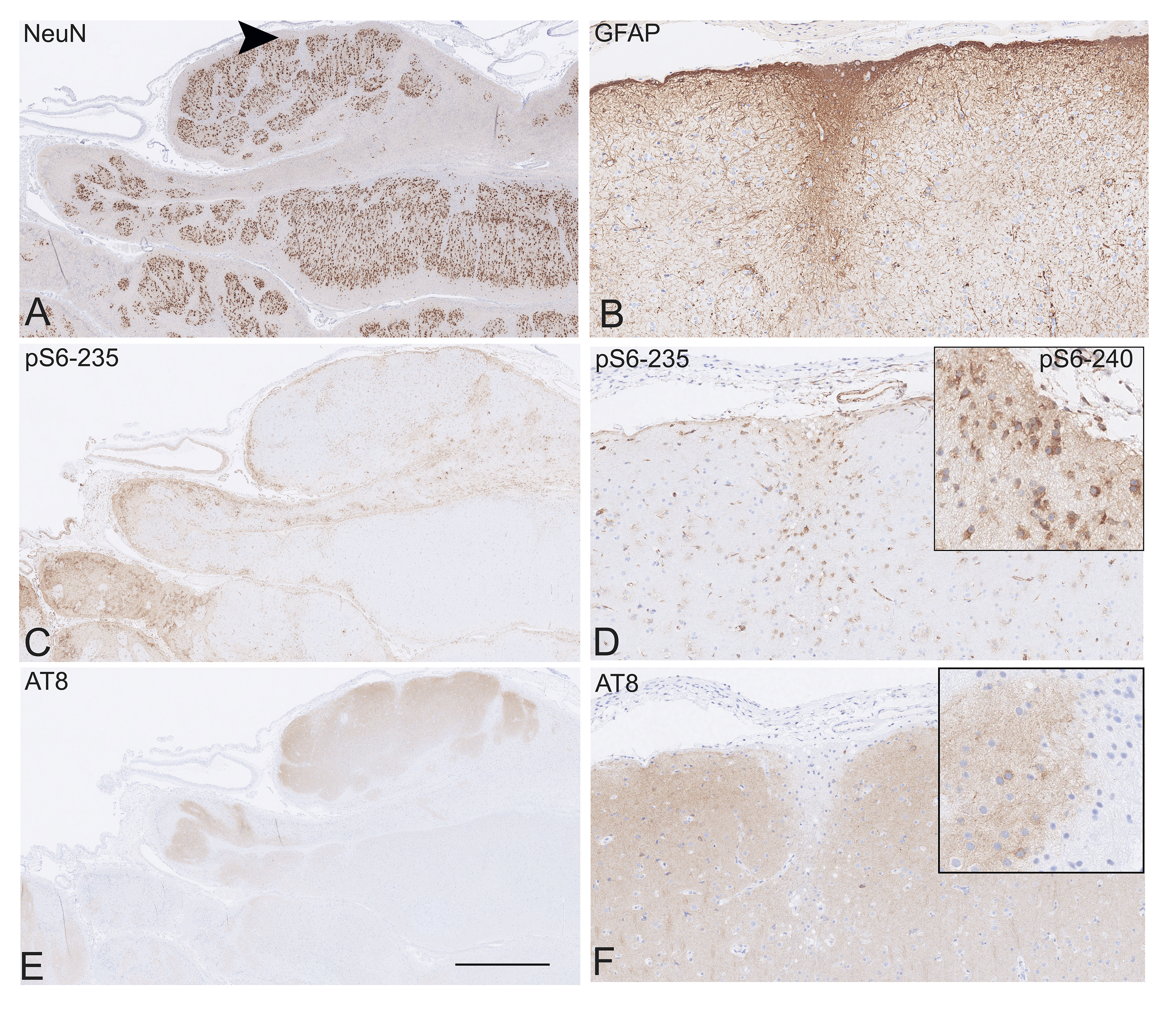

Supplement: Supplementary file 2 — Figure S1. [file EPI-66-3006-s003.zip › epi18418-sup-0011-supinfo_Supplemental_Figure_2_revised_copy.tif]
