# Supplementary material for: Patterns of phosphorylated tau accumulation in a spectrum of acquired and developmental brain lesions associated with refractory epilepsy
Source: Epilepsia. 2025 Apr 29;66(8):3006–21. doi: 10.1111/epi.18418 (PMC12371652; doi:10.1111/epi.18418)
Supplement: Supplementary file 4 — Table S2. [file EPI-66-3006-s002.docx]

| **STUDY (First author, year of publication)** | **NUMBER of cases in study** | **Epilepsy surgical pathology type** | **TAU TYPES : pTau markers, isoforms, other PTMs** |
| --- | --- | --- | --- |
| Toscana et al., 2023^1^ | 22 | TLE-HS | CP13 (Ser 202) |
| Aroor et al., 2023^2^ | 12 | TLE, HS, FCD I/III | AT8 (Ser 202, Thr205), THR 205,161 |
| Silva et al., 2021^3^ | 56 | TLE-HS or path-negative | AT8 |
| Puvena et al., 2016^4^ | 19 | TLE and FCD | AT8, CP13, TAU5 (Total Tau), Insoluble Tau |
| Tai et al., 2016^5^ | 33 | TLE-HS | AT8 |
| Prada Jardim et al. 2018^6^ | 92 | TLE-HS | AT8 |
| Jones et al., 2018^7^ | 10 | TLE, FLE | AT8 |
| Smith et al.,2019^8^ | 60 | TLE, FLE, Parietal (HS or non-lesional) | AT8 |
| Gourmaud et al., 2020^9^ | 19 | TLE-HS, FCD, DNT | AT8. AT180 (Thr 231), 4R, TAU5 |
| Hwang et al., 2023^10^ | 11 | TS (PM) | CP13, PHF1 (Ser 396-404), 3R/4R, Tau Ac (K274, K343), |
| Liu et al 2022^11^ | 10 | TS | AT8 (data shown), GT-38 (3R/4R), AV1451 (PET ligand) |
| Iyer et al., 2014^12^ | 36 | FCD type II, TS, | AT8 |
| Sen et al., 2007^13^ | 15 | FCD type II | AT8, 3R/4R |
| Kakita et al., 2005^14^ | 108 | FCD type I, II, non-lesional (temp, frontal, parietal) | AT8 |

**Supplemental Table 2. Previous studies of tau phosphorylation sites in epilepsy resections.**

Ac = acetylated tau, DNT = Dysembryoplastic neuroepithelial tumour, FCD = focal cortical dysplasia, FLE = Frontal lobe epilepsy, HS = hippocampal sclerosis, PM= post-mortem, TLE = temporal lobe epilepsy, TS= tuberous sclerosis. 3R/4R = three repeat and 4 repeat tau isoforms.

1. Toscano ECB, Vieira ELM, Grinberg LT, et al. Hyperphosphorylated Tau in Mesial Temporal Lobe Epilepsy: a Neuropathological and Cognitive Study. Mol Neurobiol 2023;60(4):2174-2185. DOI: 10.1007/s12035-022-03190-x.

2. Aroor A, Nguyen P, Li Y, Das R, Lugo JN, Brewster AL. Assessment of tau phosphorylation and beta-amyloid pathology in human drug-resistant epilepsy. Epilepsia Open 2023;8(2):609-622. DOI: 10.1002/epi4.12744.

3. Silva JC, Vivash L, Malpas CB, et al. Low prevalence of amyloid and tau pathology in drug-resistant temporal lobe epilepsy. Epilepsia 2021;62(12):3058-3067. DOI: 10.1111/epi.17086.

4. Puvenna V, Engeler M, Banjara M, et al. Is phosphorylated tau unique to chronic traumatic encephalopathy? Phosphorylated tau in epileptic brain and chronic traumatic encephalopathy. Brain Res 2016;1630:225-40. DOI: 10.1016/j.brainres.2015.11.007.

5. Tai XY, Koepp M, Duncan JS, et al. Hyperphosphorylated tau in patients with refractory epilepsy correlates with cognitive decline: a study of temporal lobe resections. Brain 2016;139(Pt 9):2441-55. DOI: 10.1093/brain/aww187.

6. Prada Jardim A, Liu J, Baber J, et al. Characterising subtypes of hippocampal sclerosis and reorganization: correlation with pre and postoperative memory deficit. Brain Pathol 2018;28(2):143-154. DOI: 10.1111/bpa.12514.

7. Jones AL, Britton JW, Blessing MM, Parisi JE, Cascino GD. Chronic traumatic encephalopathy in an epilepsy surgery cohort: Clinical and pathologic findings. Neurology 2018;90(6):e474-e478. DOI: 10.1212/WNL.0000000000004927.

8. Smith KM, Blessing MM, Parisi JE, Britton JW, Mandrekar J, Cascino GD. Tau deposition in young adults with drug-resistant focal epilepsy. Epilepsia 2019;60(12):2398-2403. DOI: 10.1111/epi.16375.

9. Gourmaud S, Shou H, Irwin DJ, et al. Alzheimer-like amyloid and tau alterations associated with cognitive deficit in temporal lobe epilepsy. Brain 2020;143(1):191-209. DOI: 10.1093/brain/awz381.

10. Hwang JL, Perloff OS, Gaus SE, et al. Tuberous sclerosis complex is associated with a novel human tauopathy. Acta Neuropathol 2023;145(1):1-12. DOI: 10.1007/s00401-022-02521-5.

11. Liu AJ, Lusk JB, Ervin J, Burke J, O'Brien R, Wang SJ. Tuberous sclerosis complex is a novel, amyloid-independent tauopathy associated with elevated phosphorylated 3R/4R tau aggregation. Acta Neuropathol Commun 2022;10(1):27. DOI: 10.1186/s40478-022-01330-x.

12. Iyer A, Prabowo A, Anink J, Spliet WG, van Rijen PC, Aronica E. Cell injury and premature neurodegeneration in focal malformations of cortical development. Brain Pathol 2014;24(1):1-17. DOI: 10.1111/bpa.12060.

13. Sen A, Thom M, Martinian L, et al. Pathological tau tangles localize to focal cortical dysplasia in older patients. Epilepsia 2007;48(8):1447-54. DOI: 10.1111/j.1528-1167.2007.01107.x.

14. Kakita A, Kameyama S, Hayashi S, Masuda H, Takahashi H. Pathologic features of dysplasia and accompanying alterations observed in surgical specimens from patients with intractable epilepsy. J Child Neurol 2005;20(4):341-50. DOI: 10.1177/08830738050200041301.
