# Supplementary material for: Patterns of phosphorylated tau accumulation in a spectrum of acquired and developmental brain lesions associated with refractory epilepsy
Source: Epilepsia. 2025 Apr 29;66(8):3006–21. doi: 10.1111/epi.18418 (PMC12371652; doi:10.1111/epi.18418)
Supplement: Supplementary file 6 [file EPI-66-3006-s001.docx]

CASE NUMBER	GROUP	LOBE	AGE AT SURGERY (OR PM)	ONSET OF EPILEPSY	DURATION OF EPILEPSY	SIDE LEFT / RIGHT	SEX	YEAR OF SURGERY	SURGICAL =SURG / PM =PM	PROLONGED EARLY CHILDHOOD CONVULSION/FS	HEAD INJURY IN HISTORY	FURTHER PATHOLOGY DETAIL/DETAIL OF BIRTH INJURY FOR PERINATAL INFARCT	FOCAL AWARE SEIZURES	FOCAL SEIZURE WITH IMPAIRED AWARENESS	GENERALISED SEIZURES	STATUS EPILEPTICUS	ILAE OUTCOME AT 2YEAR	TYPE ICE	DAYS POST INJURY FOR ICE	pS6 5364 240/44	pS6 4857 235/236	BETA AMYLOID	MULTIPLEX TAU : AT180 CP13 AT8	MULTIPLEX TAU: AT100 PHF1 AT8	DOUBLE LABELLING : PS6-235 + AT8	DOUBLE LABELLING : GFAP + AT8
1	FCD IA	OCC	6	3	3.0	L	M	2004	SURG																	
2		OCC	10	2	8.0	L	M	2005	SURG																	
3		OCC	15	7	8.0	L	M	2005	SURG																	
4		OCC	17	10	7.0	L	F	2008	SURG																	
5		OCC	3	0	3.0	R	F	2010	SURG																	
6		OCC	2	0	2.0	R	M	2011	SURG																	
7		OCC	5	2	3.0	L	F	2012	SURG																	
8		OCC	15	5	10.0	R	M	2012	SURG																	
9		OCC	25	9	16.0	L	F	2019	SURG																	
10		OCC	3	NA	NA	n.a.		2004	SURG																	
11		OCC	39	34	5.0	R	F	2021	SURG																	
12	FCDIIIA	TEMP	48	11	36.8	R	M	2003	SURG	NO	NO		YES	YES	YES	NO	2									
13		TEMP	23	9	13.9	L	F	2007	SURG	NO	NO		YES	YES	YES	NO	2									
14		TEMP	44	13	31.1	L	M	2007	SURG	NO	NO		YES	YES	YES	YES	1			YES	YES					
15		TEMP	57	12	44.7	R	F	2009	SURG	NO	NO		NO	YES	NO	NO	1									
16		TEMP	49	9	39.9	L	F	2015	SURG	NO	YES		NO	YES	YES	YES	1									
17	FCDIIIB	TEMP	27	0.83	26.5	L	F	2011	SURG	NO	NO		YES	YES	YES	NO	1			YES	YES					
18		TEMP	23	6	16.9	R	M	2004	SURG	NO	YES		YES	YES	YES	NO	1			YES	YES					
19		TEMP	38	1	37.3	R	M	1994	SURG	NO	NO		NO	YES	YES	YES	3									
20		TEMP	42	9	32.9	L	F	2007	SURG	NO	YES		YES	YES	NO	NO	4									
21		TEMP	37	16	20.6	R	F	2002	SURG	NO	NO		YES	YES	NO	NO	1									
22		TEMP	41	32	8.6	L	M	2012	SURG	NO	YES		NO	YES	NO	NO	1									
23	CAVERNOMA	TEMP	60	60	0.3	L	F	2014	SURG																	
24		TEMP	49	31	18.2	R	M	2011	SURG	NO	NO		YES	YES	YES	NO	4					YES			YES	YES
25		PAR	40	6	33.6	L	M	2013	SURG	YES	NO		YES	YES	NO	NO	4									
26		FRONT	36	31	5.5	R	M	2013	SURG	NO	NO		NO	YES	YES	YES	4									
27		TEMP	27	23	3.6	L	F	2010	SURG	NO	NO		NO	YES	NO	NO	4									
28		TEMP	34	26	7.5	L	M	2019	SURG	NO	YES		YES	YES	YES	NO	1									
29		TEMP	62	52	10.4	L	M	2019	SURG	NO	NO		YES	YES	YES	NO	1									
30		TEMP	43	31	11.6	R	M	2011	SURG	NO	NO		NO	YES	YES	NO	1					YES	YES	YES	YES	YES
31		TEMP	51	46	5.0	L	M	2009	SURG																	
32		TEMP	63	36	26.7	R	M	2023	SURG	NO	NO		YES	YES	YES	NO						YES	YES	YES	YES	YES
33		TEMP	68	61	6.5	L	F	2013	SURG	NO	NO		NO	YES	NO	NO	1									
34	STURGE-WEBER SYNDROME	OCC	47	40	6.6	L	F	2021	SURG	NO	NO		YES	YES	NO	NO						YES			YES	YES
35		OCC	59	8	51.2	R	F	2014	SURG	NO	NO		YES	YES	YES	NO	4					YES	YES	YES	YES	YES
36		TEMP	43	18	25.0	R	M	2013	SURG																	
37		FRONT	6	2	4.0	L	M	2013	SURG																	
38			44	NA	NA	NA	M	2014	SURG																	
39		FRONT	1	0.5	0.5	R	M	2015	SURG																	
40			1	NA	NA	R	M	2016	SURG																	
41		TEMP	9	5	4.0	R	F	2016	SURG																	
42		PAR	16	10	6.0	L	M	2018	SURG																	
43		FRONT	2	NA	NA	L	M	2022	SURG																	
44	MENINGIO-ANGIO-MATOSIS	TEMP	35	12	22.7	L	M	2001	SURG	NO	YES		YES	YES	YES	NO	1					YES	YES	YES	YES	YES
45		FRONT	67	67	0.5	R	M	1994	SURG					YES												
46		TEMP	61	21	39.5	R	F	2015	SURG	NO	YES		NO	YES	YES	NO	1									
47		TEMP	54	18	35.6	R	F	2013	SURG	NO	NO		NO	YES	YES	NO	1									
48	FCDIIID WITH PERINATAL INFARCT	OCC	23	11	12.0	L	M	2014	SURG	NO	NO	NIS : PROLONGED LABOUR	YES	YES	NO	NO										
49		HEM	37	2	34.5	L	F	1998	SURG	NO	NO	PPIS	YES	YES	YES	NO	3									
50		FRONT	30	18	11.7	L	M	2015	SURG	NO	NO	PPIS	YES	YES	YES	NO	4									
51		OCC	26	12	13.9	R	M	2023	SURG	NO	NO	NIS : CHD	YES	YES	YES	NO				YES	YES	YES			YES	YES
52		TEMP	2	NA	NA			2004	SURG			PPIS														
53		TEMP	8	PAED	8.0	L	F	2014	PM	NO		PPIS														
54		OCC	52	PAED		BILAT	M	2013	PM			PPIS														
55		OCC	27	15	12.0	R	M	1997	SURG	NO	NO	PPIS	YES	NO	YES	NO				YES	YES	YES				
56		OCC	50			BILAT	F	2024	PM			NIS : BIRTH INJURY													YES	
57	FCD IIID / RE	HEM	17	1.5	15.5	R	M	1994	SURG	NO	NO		YES	YES	YES	YES	1									
58		HEM	19	14	4.8	L	F	1997	SURG	NO	NO		YES	NO	YES	YES	1			YES						
59		HEM	15			R	F	1995	SURG					YES												
60		TEMP	24	17	6.9	R	F	2008	SURG	NO	NO		NO	YES	YES	YES	5									
61		OCC	57	21	36.0	BILAT	F	2021	PM	NO	NO	END STAGE RE	YES	YES	YES	YES	5									
62		TEMP	37	17	20.0	BILAT	F	2023	PM			ACTIVE RE			YES	YES										
63	GM HETEROTOPIA	TEMP	69	NA	33.0	BILAT	F	2003	PM			PVNH AND AREA OF PMG														
64		PAR	69	19	50.0	BILAT	F	2022	PM		YES	LAMINAR HETEROTOPIA		YES	YES	YES										
65		PAR	83	3	80.0	BILAT	M	1994	PM		NO	LAMINAR HETEROTOPIA														
66		TEMP	25	2	23.0	BILAT	M	1995	PM			LAMINAR + COMPLEXT MCD		YES	YES											
67		HEM	37	10	27.0	BILAT	F	1991	PM		YES	LAMINA R HETEROTOPIA		YES	YES											
68		FRONT	51	22	29.0	BILAT	F	1994	PM		YES	PVNH		YES	YES											
69	INTRACRANIAL RECORDING SCARS	TEMP	24	0.4	7.2	R	F	2012	SURG	YES	NO	OLD SCAR AND HS	YES	YES	YES	NO	3	GRID	5							
70		TEMP	30	15	15.0	L	F	2009	SURG	NO	NO	HS TYPE 1	NO	YES	YES	NO	5	GRID	8							
71		PAR	19	7	11.8	R	F	2008	SURG	NO	NO	FCD 2A	YES	YES	YES	YES	4	GRID AND TRACK	8	YES	YES					
72		FRONT	39	7	32.2	R	M	2012	SURG	NO	NO	PATHOLOGY NEG	NO	YES	YES	NO	5	GRID	9	YES	YES					
73		TEMP	24	15	8.6	L	F	2009	SURG	NO	NO	PATH OLOGY NEG	NO	YES	YES	NO	5	TRACK	10	YES						
74		FRONT	35	7	27.8	L	F	2011	SURG	NO	NO	FCD 2B	YES	YES	YES	YES	1	TRACK	10	YES	YES					
75		FRONT	31	18	12.7	R	M	2012	SURG	NO	NO	PATHOLOGY NEG	YES	YES	YES	NO	4	GRID	10	YES	YES					
76		PAR	32	12	20.2	R	M	2011	SURG	NO	NO	FCD2B	YES	YES	YES	NO	1	GRID AND TRACK	11							
77		FRONT	40	27	13.4	R	M	2010	SURG	NO	NO	FCD2B	YES	YES	YES	NO	1	GRID	11							
78		FRONT	30	12	17.6	L	M	2011	SURG	NO	NO	FCD 2B	YES	NO	YES	NO	1	GRID	13	YES	YES					
79		FRONT	60	17	42.8	R	F	2010	SURG	NO	YES	MILD MCD	YES	YES	YES	NO	4	TRACK WM	28							
80		TEMP	22	14	8.2	R	F	2011	SURG	NO	NO	HS TYPE 1	NO	YES	YES	NO	3	TRACK	30							
81		FRONT	31	8	23.1	R	F	2011	SURG	NO	NO	FCD 2B	YES	YES	YES	NO	3	GRID AND TRACK	30							
82		TEMP	35	20	15.2	L	F	2011	SURG	NO	NO	HS TYPE 3	NO	YES	YES	NO	1	TRACK WM	70							
83		TEMP	26	4	22.0	R	M	2006	SURG	NO	NO	FCD 2B	NO	YES	YES	NO	2	TRACK WM	209	YES	YES					
84		FRONT	33	13	19.6	R	M	2013	SURG	NO	YES	PATHOLOGY NEG	NO	YES	NO	NO	1	TRACK WM	211							
85		FRONT	53	30	23.2	L	F	2012	SURG	NO	NO	DNT	NO	YES	YES	NO	4	TRACK CORTEX	232							
86		TEMP	34	8	26.1	L	M	2002	SURG	YES	NO	HS TYPE 1	NO	YES	YES	YES	3	TRACK WM	301							
87		TEMP	48	4	43.8	L	M	2007	SURG	NO	YES	HS TYPE 1	NO	YES	YES	NO	4	TRACK WM	329							
88	SCARS	TEMP	22	11	10.7	R	F	1995	SURG	YES	NO	SCAR FROM PREVIOUS SURGERY	YES	YES	YES	NO	5			YES	YES					
89		TEMP	28	10	17.7	R	F	2023	SURG	NO	NO	NO HISTORY OF TRAUMA	YES	YES	YES	NO										
90		FRONT	36	29	7.0	R	M	1995	SURG		YES	SCAR FROM PREVIOUS SURGERY	YES	YES	YES											
91		FRONT	43	18	25.3	R	M	2015	SURG	NO	NO	S CAR FOLLOWING PREVIOUS ICH	NO	YES	YES	NO	3									
92		FRONT	54	9	45.3	R	M	2014	SURG	NO	YES	TBI AND ABSCESS	NO	YES	YES	NO	3					YES	YES	YES	YES	YES
93		TEMP	44	35	9.4	R	M	2010	SURG	NO	NO	PREVIOUS SURGERY FOR EPIDERMOID CYST	YES	YES	YES	NO	5									
94		TEMP	31	16	15.0	L	F	2010	SURG	NO	NO	PREVIOUS ABSCESS	YES	YES	YES	YES	1									
95		TEMP	43	20	22.9	R	F	2008	SURG	NO	NO	SCAR FROM PREVIOUS SURGERY	NO	YES	YES	YES	4									
96		TEMP	39	18	20.9	R	F	2007	SURG	NO	NO	SCAR FROM PREVIOUS SURGERY	NO	YES	NO	NO	1									
97		HEM	17	3	14.0	R	F	1996	SURG	YES		SCAR FOLLOWING MENINGITIS/HYPOXIA								YES	YES					
98	ENCEPHALOCELE	TEMP	32	5	26.9	R	F	2021	SURG	NO	YES		NO	YES	YES	NO										
99		TEMP	45	22	23.4	R	F	2018	SURG	NO	NO		YES	YES	YES	NO	1									
100		TEMP	50	25	24.9	L	F	2017	SURG	NO	NO		NO	YES	NO	NO	2									
101		TEMP	47	19	28.2	L	F	2023	SURG	NO	YES		YES	YES	YES	NO						YES	YES	YES	YES	YES
102		TEMP	28	12	16.3	L	M	2023	SURG	NO	YES		YES	YES	NO	NO										
103		TEMP	36	25	6.0	R	F	2018	SURG			ADDITIONAL MMCD	YES	YES	YES											
104		TEMP	47	18	29.0	L	M	2023	SURG	NO	NO		YES		YES											

Supplemental Table 1. Detail of epilepsy, clinical history and additional stains in the 12 pathology groups.Supplemental Table 1. Detail of epilepsy, clinical history and additional stains in the 12 pathology groups.

CHD = congenital heart disease, SURG=surgical tissue sample, HEM = hemispherectomy and when sample site not specified, CHD = congenital heart disease, FRONT= frontal lobe, GM= grey matter, FCD = focal cortical dysplasia, F=female, GRID = from surface grid cortical injury, HS = Hippocampal sclerosis, ICE = intracranial electrode injury, MMCD = Mild malformation of cortical development, M= male, NA= not available, NIS = neonatal ischaemic stroke, OCC= occipital, PM= post-mortem tissue sample, PPIS = presumed perinatal ischaemic stroke, Track = from electrode track scar, RE = Ramussen's encephalitis, TBI= Traumatic brain injury, TEMP= temporal lobe including media structures, WM= white matter.
